# Lifetime Estimation for Multi-Phase Deteriorating Process with Random Abrupt Jumps †

**DOI:** 10.3390/s19061472

**Published:** 2019-03-26

**Authors:** Jianxun Zhang, Xiaosheng Si, Dangbo Du, Chen Hu, Changhua Hu

**Affiliations:** 1 Department of Automation, Xi’an Research Institute of High-Tech, Xi’an 710025, China; jx-zhang14@tsinghua.org.cn (J.Z.); ddb_effort@126.com (D.D.); chenh628@hotmail.com (C.H.); 2 The Key Laboratory of Education Ministry for Modern Design and Rotor-Bearing System, Xi’an Jiaotong University, Xi’an 710049, China

**Keywords:** life prognostics, reliability, multi-phase degradation, expectation maximization algorithm, random jump

## Abstract

Owing to operating condition changing, physical mutation, and sudden shocks, degradation trajectories usually exhibit multi-phase features, and the abrupt jump often appears at the changing time, which makes the traditional methods of lifetime estimation unavailable. In this paper, we mainly focus on how to estimate the lifetime of the multi-phase degradation process with abrupt jumps at the change points under the concept of the first passage time (FPT). Firstly, a multi-phase degradation model with jumps based on the Wiener process is formulated to describe the multi-phase degradation pattern. Then, we attain the lifetime’s closed-form expression for the two-phase model with fixed jump relying on the distribution of the degradation state at the change point. Furthermore, we continue to investigate the lifetime estimation of the degradation process with random effect caused by unit-to-unit variability and the multi-phase degradation process. We extend the results of the two-phase case with fixed parameters to these two cases. For better implementation, a model identification method with off-line and on-line parts based on Expectation Maximization (EM) algorithm and Bayesian rule is proposed. Finally, a numerical case study and a practical example of gyro are provided for illustration.

## 1. Introduction

With the rapid development of technology, prognostics and health management (PHM) technique has gained increasing attention and been applied to industrial systems, military devices, aerospace equipment, and so on [1,2]. As the key to PHM technique, the lifetime or remaining useful lifetime (RUL) estimation approach can provide effective information for maintenance policy decision, and thus it has attracted much attention in recent years [3,4,5,6]. Generally speaking, the lifetime estimation approach can be classified into the categories of physics of failure, data-driven and fusion [6]. However, it is not easy to make clear the degradation mechanism and build the mechanism model for complex deteriorating systems. As such, the data-driven approach has gained momentum owing to its good feasibility [3,6,7,8,9]. As discussed in [6], Jardine points out that the stochastic data-driven approach can efficiently describe the degradation trajectories’ uncertainty and randomness, since it just relies on observed data and the statistical process model. Nowadays, the stochastic data-driven approach has been widely investigated and attracted more attention, such as in the Wiener-process-based model [10,11], the Gamma-process-based model [12], the Inverse-Gaussian-process-based model [13,14], and so on. In this paper, we mainly concentrate on how to estimate the lifetime under the concept of the first passage time (FPT) based on the stochastic data-driven model.

In practice engineering, due to operating condition changing, physical mutation, and sudden shock, the degradation patterns of some systems cannot remain unchanged during their whole life cycles, and their degradation process often exhibit multi-phase feature, such as plasma display panels (PDPs) [15], light emitting diode (LED) [16], liquid crystal display (LCD) [17], battery [18], and so on. For example, Burgess [19] pointed out that the battery’s capacity fade process should be divided into two stages: a slowly fading process and a much more rapidly fading process. Therefore, the traditional model with single phase may not be suitable for the multi-phase degradation process, and thus the multi-phase model should be taken into account. So far, some multi-phase degradation models have been proposed. In 2008, Ng [20] proposed an independent-increments stochastic mode based on Poisson process with a change point for two-phase degradation trajectory. Bae et al., [15,21] proposed a two-phase regression model and a change point detection approach to deal with the degradation of plasma display. To predict bearing’s RUL, Wang et al., [22] proposed an adaptive RUL estimation method for the two-phase degradation model based on enhanced Kalman filter and the EM algorithm. Wang et al., [17] and Yan et al., [16] proposed a reliability evaluation method for LCD based on the two-phase Wiener process. Recently, Wang et al., [23,24] utilized the Bayesian rule and change-point Wiener process to model the degradation process of PDPs and LED, and Zhang et al., [25] attained some analytical results of lifetime’s distribution based on the two-phase Wiener process.

To the best of our knowledge, most aforementioned research mainly focuses on the continuous multi-phase degradation process ignoring the abrupt jump. However, the abrupt jump often appears at the change point owing to the condition changing or physical mutation and only a few works refers to this issue [26,27]. Kong et al. [26] utilized a two-phase Wiener process model with an abrupt fixed jump to model the bearing’s degradation, and further proposed a method of lifetime estimation. Unfortunately, the abrupt jump is defined as a fixed value and the diffusion coefficient does not change at different phases, which may limit its application. In addition, the degradation feature of different deteriorating systems (e.g., the degradation rate and the jump amplitude) may not be the same owing to the heterogeneity. Therefore, if parameters of the degradation model are defined as the fixed value, the heterogeneity of a batch of systems cannot be reflected. Besides, the degradation state at the changing time is unknown until the change point appears due to the degradation’s randomness, and it should be a random variable determined by both the time at the change point and the degradation model of the first phase. To describe the randomness at the change point and reduce the calculation complexity, some researchers used the mean of the state at the change point replacing its actual distribution for lifetime estimation such as [17,28]. But it may lead to estimate bias.

We attempt to deal with these problems from the perspective of the stochastic process and statistic analysis. The contributions of the paper can be summarized as follows: (1) We propose a two-phase degradation model with the fixed jump at the change point governed by the Wiener process, and then attain the analytical form of the lifetime under the concept of FPT with a predefined changing time and fixed parameters. (2) We extend the results of the two-phase model with fixed parameters to the multi-phase case and the case with random effect. In these two cases, we obtain the lifetime’s expression with a multi-integral form. (3) We further provide a model identification method based on the EM algorithm and Bayesian rule. (4) Finally, to illustrate the applicability and effectiveness of our method, a numerical example and a practical case of the gyro are provided.

The remainder of the paper is organized as follows. In Section 2, the motivating example and problem formulation are introduced, and a general multi-phase degradation model with random jumps is formulated. Section 3 includes the main results of lifetime estimation. In Section 4, realization of parameter identification is explained. Two illustrative examples are presented to illustrate the proposed model in Section 5. This paper is concluded in Section 6.

## 2. Motivation and Problem Formulation

**Figure 1:**
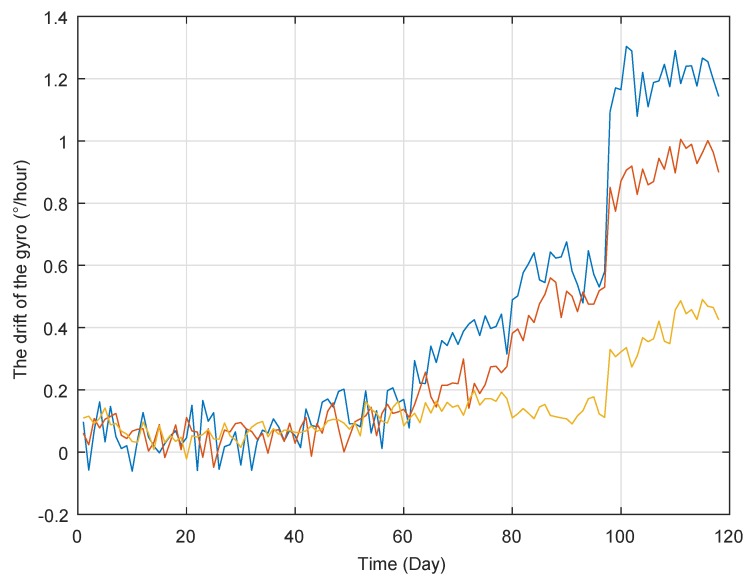
The degradation trajectories of the gyros.

It is well-known that gyro is a typical sensor used for measuring the speed and acceleration, which has widely been applied into vessel, airplane, aircraft, and so on. However, its performance will deteriorate over time, which may decrease the positioning accuracy. Figure 1 shows the degradation data of three gyros collected from a step-stress accelerated degradation testing. The degradation data are collected from many experiments, and the duration time is about one day for each experiment. Therefore, the unit of X-axis is set as “day” rather than “times”. In Figure 1, it could be found that there are obvious abrupt jumps in their degradation trajectories of three gyros’s drift, and they can be classified into two stages with different rates. It is interesting to see that the changing time is also the stress switch time, which means that the operating condition switch changes the degradation feature. In addition, as discussed in [26,29], the degradation process of bearing also exhibits two-phase feature and the abrupt jump exists at the change point. That is to say, the degradation trajectory could not be described well by the traditional single-stage degradation model. Therefore, it is natural to model these degradation processes based on the multi-phase model with an abrupt jump.

From Figure 1 and the bearing’s degradation process in [26,29], the degradation trajectories are non-monotone. Thus, it is natural to adopt the Wiener process to describe these non-monotone degradation. To simplify the problem, the following Assumption 1 is given,

**Assumption** **1.** *The abrupt jump at the change point is instantaneous. That is to say, if the changing time and the jump are defined as τ and γ separately, the abrupt jump does not appear until t=τ, and the degradation process will increase or decrease γ suddenly at the time t=τ.*

Inspired by Kong’ method [26], we provide a two-phase Wiener process with random jump as follows,

(1) $$\begin{array}{c} X\left(t\right)=\left\{\begin{array}{cc} x_{0}+\mu_{1}t+\sigma_{1}^{}B\left(t\right), & 0<t<\tau \\ x_{\tau }+\mu_{2}\left(t-\tau \right)+\sigma_{2}^{}B\left(t-\tau \right), & t\geq \tau \end{array}\right. \end{array}$$

where $\mu_{1}$ and $\mu_{2}$ are the drift coefficients reflecting the degradation rate, $\sigma_{1}$ and $\sigma_{2}$ are the diffusion coefficients reflecting the uncertainty of the degradation process, $x_{0}$ denotes the initial value of the degradation process, τ is the changing time and $\tau^{-}$ denotes its left limit, B(t) is the standard Brown Motion, $x_{\tau }\;=\;x_{\tau^{-}}\;+\;\gamma$ is the initial value of the second phase degradation, and γ is the random jump. Then, we can extend (1) to the multiple case simply.

(2) $$\begin{array}{c} X\left(t\right)=\left\{\begin{array}{cc} x_{0}+\mu_{1}t+\sigma_{1}^{}B\left(t\right), & 0<t<\tau_{1}\\ \vdots & \\ x_{\tau_{i}}+\mu_{i+1}\left(t-\tau_{i}\right)+\sigma_{i+1}^{}B\left(t-\tau_{i}\right), & \tau_{i-1}<t<\tau_{i}\\ \vdots & \\ x_{N}+\mu_{N+1}\left(t-\tau_{i}\right)+\sigma_{N+1}^{}B\left(t-\tau_{N}\right), & \tau_{N}<t \end{array}\right. \end{array}$$

where $x_{\tau_{i}}$ denotes the initial of the (i+1)-th phase, $\mu \;=\;[\mu_{1},\mu_{2},\ldots ,\mu_{N}]$ and $\sigma \;=\;[\sigma_{1},\sigma_{2},\ldots ,\sigma_{N}]$ represent all drift and diffusion coefficients at each phase, and $\gamma \;=\;[\gamma_{1},\gamma_{2},\ldots ,\gamma_{N}]$ is the jump at each change points.

In this paper, we concentrate on how to derive the lifetime distribution under the concept of first passage time (FPT). Generally, the lifetime under the concept of FPT is a random variable and it is usually defined as follows,

(3) $$\begin{array}{c} T:=\inf\{t:X\left(t\right)\geq \xi \left|X(0)\leq \xi \right.\} \end{array}$$

where ξ represents the threshold which should be predefined in our method, *T* is FPT of the degradation process and it also represents the lifetime with probability density function (PDF) $f_{T}\left(t\right)$ and cumulative distribution function (CDF) $F_{T}\left(t\right)$ in this paper. Similar to the definition of lifetime, we can further provide the expression of remaining useful life (RUL) under the concept of FPT as follows,

(4) $$\begin{array}{c} L_{k}:=\inf\left\{l_{k}:X\left(t_{k}+l_{k}\right)\geq \xi \left|X(t_{k})\leq \xi \right.\right\} \end{array}$$

where $l_{k}$ denotes the RUL with PDF $f_{l_{k}}\left(t\right)$ and CDF $F_{l_{k}}\left(t\right)$ at the time $t_{k}$.

## 3. Lifetime Estimation under the Concept of the FPT

### 3.1. Lifetime Estimation for Two-Phase Degradation Process without Random Effect

Firstly, we consider a simple case i.e., two-phase degradation model with fixed parameters as shown in (1). As discussed in [16,25], if the abrupt jump is equal to 0 and $x_{\tau }$ is given, the expression of its lifetime distribution can be obtained as follows,

(5) $$\begin{array}{c} f_{T}\left(t\right)=\left\{\begin{array}{cc} \frac{\xi -x_{0}}{\sqrt{2\pi \sigma_{1}^{2}t^{3}}}\exp\left[-\frac{{\left(\xi -x_{0}-\mu_{1}t\right)}^{2}}{2\sigma_{1}^{2}t}\right], & 0<t\leq \tau \\ \frac{\xi -x_{\tau }}{\sqrt{2\pi \sigma_{2}^{2}{\left(t-\tau \right)}^{3}}}\exp\left[-\frac{{\left(\xi -x_{\tau }-\mu_{2}\left(t-\tau \right)\right)}^{2}}{2\sigma_{2}^{2}\left(t-\tau \right)}\right], & t>\tau \end{array}\right. \end{array}$$

However, unlike the two-phase model without abrupt jump, the lifetime of the two-phase model with jump is not continuous at the change point. It is noteworthy that we should first derive the form of $x_{\tau^{-}}$ relying on $x_{\tau }\;=\;x_{\tau^{-}}+\gamma$. Unfortunately, if the change point does not appear, the value of $x_{\tau^{-}}$ should be a random variable rather a fixed value. Besides, it is determined by the degradation model at the first phase and the time of the change point.

To obtain the lifetime under the concept of FPT, we should derive the expression of $x_{\tau^{-}}$ under the concept of FPT, i.e., under the condition T>τ. It is defined that $g_{\tau^{-}}\left(x_{\tau^{-}}\right)$ denotes the PDF of $x_{\tau^{-}}$ under the concept of FPT. Based on the result of our previous work [25], $g_{\tau^{-}}\left(x_{\tau^{-}}\right)$ has the following analytical form,

(6) $$\begin{array}{c} g_{\tau^{-}}\left(x_{\tau^{-}}\right)=\frac{1}{\sqrt{2\pi \tau^{-}\sigma_{1}^{2}}}\left\{\exp\left[-\frac{{\left(x_{\tau^{-}}-\mu_{1}\tau^{-}\right)}^{2}}{2\sigma_{1}^{2}\tau^{-}}\right]\right.\left.-\exp\left(\frac{2\mu_{1}\xi }{\sigma_{1}^{2}}\right)\exp\left[-\frac{{\left(x_{\tau^{-}}-2\xi -\mu_{1}\tau^{-}\right)}^{2}}{2\sigma_{1}^{2}\tau^{-}}\right]\right\} \end{array}$$

where $\tau^{-}\;=\;\tau$ due to the continuity of time, and the range of $x_{\tau^{-}}$ is (−∞,ξ) owing to the definition of FPT. Moreover, to facilitate calculation, the following Lemma 1 is provided,

**Lemma** **1.** *[25]: If y is a Gaussian random variable following $N(\mu_{b},\sigma_{b}^{2})$, then $\exp\left[-\frac{{\left(y\;-\;\mu_{a}\right)}^{2}}{2\sigma_{a}^{2}}\right]$ and $y\exp\left[-\frac{{\left(y\;-\;\mu_{a}\right)}^{2}}{2\sigma_{a}^{2}}\right]$ hold the following forms,*

(7) $$\begin{array}{c} \int_{-\infty }^{\xi }\exp\left[-\frac{{\left(y-\mu_{a}\right)}^{2}}{2\sigma_{a}^{2}}\right]\frac{1}{\sqrt{2\pi \sigma_{b}^{2}}}\exp\left[-\frac{{\left(y-\mu_{b}\right)}^{2}}{2\sigma_{b}^{2}}\right]dy\\ =\sqrt{\frac{\sigma_{a}^{2}}{\left(\sigma_{a}^{2}+\sigma_{b}^{2}\right)}}\exp\left(-\frac{{\left(\mu_{a}-\mu_{b}\right)}^{2}}{2\left(\sigma_{a}^{2}+\sigma_{b}^{2}\right)}\right)\left[1-\Phi \left(-\frac{\xi \left(\sigma_{a}^{2}+\sigma_{b}^{2}\right)-\mu_{b}\sigma_{a}^{2}-\mu_{a}\sigma_{b}^{2}}{\sqrt{\sigma_{a}^{2}\sigma_{b}^{2}\left(\sigma_{a}^{2}+\sigma_{b}^{2}\right)}}\right)\right] \end{array}$$

and,

(8) $$\begin{array}{c} \begin{array}{c} \int_{-\infty }^{\xi }y\exp\left[-\frac{{\left(y-\mu_{a}\right)}^{2}}{2\sigma_{a}^{2}}\right]\frac{1}{\sqrt{2\pi \sigma_{b}^{2}}}\exp\left[-\frac{{\left(y-\mu_{b}\right)}^{2}}{2\sigma_{b}^{2}}\right]dy\\ =\sqrt{\frac{\sigma_{a}^{2}}{\left(\sigma_{a}^{2}+\sigma_{b}^{2}\right)}}\exp\left(-\frac{{\left(\mu_{a}-\mu_{b}\right)}^{2}}{2\left(\sigma_{a}^{2}+\sigma_{b}^{2}\right)}\right)\times \\ \left[\frac{\mu_{b}\sigma_{a}^{2}+\mu_{a}\sigma_{b}^{2}}{\sigma_{a}^{2}+\sigma_{b}^{2}}\Phi \left(\frac{\xi \left(\sigma_{a}^{2}+\sigma_{b}^{2}\right)-\mu_{b}\sigma_{a}^{2}-\mu_{a}\sigma_{b}^{2}}{\sqrt{\sigma_{a}^{2}\sigma_{b}^{2}\left(\sigma_{a}^{2}+\sigma_{b}^{2}\right)}}\right)-\sqrt{\frac{\sigma_{a}^{2}\sigma_{b}^{2}}{\sigma_{a}^{2}+\sigma_{b}^{2}}}\phi \left(\frac{\xi \left(\sigma_{a}^{2}+\sigma_{b}^{2}\right)-\mu_{b}\sigma_{a}^{2}-\mu_{a}\sigma_{b}^{2}}{\sqrt{\sigma_{a}^{2}\sigma_{b}^{2}\left(\sigma_{a}^{2}+\sigma_{b}^{2}\right)}}\right)\right] \end{array} \end{array}$$

Then, we can obtain the lifetime’s distribution as shown in followings,

(9) $$f_{T}\left(t\right)=\frac{\xi -x_{0}}{\sqrt{2\pi \sigma_{1}^{2}t^{3}}}\exp\left[-\frac{{\left(\xi -x_{0}-\mu_{1}t\right)}^{2}}{2\sigma_{1}^{2}t}\right],\;0<t<\tau$$

(10) $$\begin{array}{c} \begin{array}{c} f_{T}\left(t\right)=\sqrt{\frac{1}{2\pi {\left(t-\tau \right)}^{2}\left(\sigma_{a1}^{2}+\sigma_{b}^{2}\right)}}\exp\left[-\frac{{\left(\mu_{a1}-\mu_{b1}\right)}^{2}}{2\left(\sigma_{a1}^{2}+\sigma_{b1}^{2}\right)}\right]\\ \times \left\{\frac{\mu_{b1}\sigma_{a1}^{2}+\mu_{a1}\sigma_{b1}^{2}}{\sigma_{a1}^{2}+\sigma_{b1}^{2}}\Phi \left(\frac{\mu_{b1}\sigma_{a1}^{2}+\mu_{a1}\sigma_{b1}^{2}}{\sqrt{\sigma_{a1}^{2}\sigma_{b1}^{2}\left(\sigma_{a1}^{2}+\sigma_{b1}^{2}\right)}}\right)+\sqrt{\frac{\sigma_{a1}^{2}\sigma_{b1}^{2}}{\sigma_{a1}^{2}+\sigma_{b1}^{2}}}\phi \left(\frac{\mu_{b1}\sigma_{a1}^{2}+\mu_{a1}\sigma_{b1}^{2}}{\sqrt{\sigma_{a1}^{2}\sigma_{b1}^{2}\left(\sigma_{a1}^{2}+\sigma_{b1}^{2}\right)}}\right)\right\}\\ -\exp\left(\frac{2\mu_{1}\xi }{\sigma_{1}^{2}}\right)\sqrt{\frac{1}{2\pi {\left(t-\tau \right)}^{2}\left(\sigma_{a1}^{2}+\sigma_{b1}^{2}\right)}}\exp\left[-\frac{{\left(\mu_{a1}-\mu_{c1}\right)}^{2}}{2\left(\sigma_{a1}^{2}+\sigma_{b1}^{2}\right)}\right]\\ \times \left\{\frac{\mu_{c1}\sigma_{a1}^{2}+\mu_{a1}\sigma_{b1}^{2}}{\sigma_{a1}^{2}+\sigma_{b1}^{2}}\Phi \left(\frac{\mu_{c1}\sigma_{a1}^{2}+\mu_{a1}\sigma_{b1}^{2}}{\sqrt{\sigma_{a1}^{2}\sigma_{b1}^{2}\left(\sigma_{a1}^{2}+\sigma_{b1}^{2}\right)}}\right)+\sqrt{\frac{\sigma_{a1}^{2}\sigma_{b1}^{2}}{\sigma_{a1}^{2}+\sigma_{b1}^{2}}}\phi \left(\frac{\mu_{c1}\sigma_{a1}^{2}+\mu_{a1}\sigma_{b1}^{2}}{\sqrt{\sigma_{a1}^{2}\sigma_{b1}^{2}\left(\sigma_{a1}^{2}+\sigma_{b1}^{2}\right)}}\right)\right\} \end{array} \end{array}$$

where

(11) $$\begin{array}{c} \begin{array}{c} \mu_{a1}=\mu_{2}\left(t-\tau \right),\mu_{b1}=\xi -\mu_{1}\tau ,\mu_{c1}=-\xi -\mu_{1}\tau \\ \sigma_{a1}^{2}=\sigma_{2}^{2}\left(t-\tau \right),\sigma_{b1}^{2}=\sigma_{1}^{2}\tau , \end{array} \end{array}$$

and

(12) $$\begin{array}{ccc} \mathrm{Pr}\{T=\tau \} & = & \mathrm{Pr}\{x_{\tau }=x_{\tau^{-}}+\gamma \geq \xi \}\\ & = & I\left(\gamma \leq 0\right)\cdot 0+I\left(\gamma >0\right)\left[\Phi \left(\frac{\mu_{1}t-\xi }{\sigma \sqrt{t}}\right)-\exp\left(\frac{2\xi \mu_{1}}{\sigma }\right)\Phi \left(\frac{-\mu_{1}t-\xi }{\sigma \sqrt{t}}\right)\right]\\ & & +I\left(\gamma >0\right)\left[-\Phi \left(\frac{\mu_{1}t-\xi +\gamma }{\sigma \sqrt{t}}\right)+\exp\left(\frac{2\left(\xi -\gamma \right)-\mu_{1}}{\sigma_{1}}\right)\Phi \left(\frac{-\mu_{1}t-\xi +\gamma }{\sigma_{1}\sqrt{t}}\right)\right] \end{array}$$

where I(·) is the indicator function. It is noteworthy that if the value of the abrupt jump is negative (γ<0), the failure probability at T=τ is 0, which means the degradation process will not pass the given threshold suddenly at the changing time. On the contrary, the failure probability at T=τ is larger than 0, which means that the degradation may pass the threshold and lead to sudden failure of deteriorating device.

**Proof.**  See Appendix A. □

In this way, the lifetime distribution at 0≤T<τ, T=τ, and T>τ, have been derived i.e., (9), (12), and (10).

**Remark** **1.** *It is noteworthy that due to the effect of the abrupt jump at the change point, the degradation process may pass the given failure threshold suddenly. That is to say, the distribution of the lifetime is not continuous at the changing time. Thus, the lifetime’s distribution is described separately at three different cases as shown in (9), (12), and (10).*

### 3.2. Lifetime Estimation for Two-Phase Degradation Model with Random Effect

In practical engineering, the parameters of different deteriorating systems may be different, which is caused by the unit-to-unit variability of the degradation systems. Inspired by the literature [30], the random effect on the degradation process is adopted to describe the difference between the different devices.

**Assumption** **2.** *To reflect such the random effect, we assume that the parameters are defined as the random variable rather than deterministic parameters. Similar to the definitions in [16,20,30], in this subsection, it is assumed that $\mu_{1}$, $\mu_{2}$, and γ follow Gaussian distribution with mean $\mu_{1p}$, $\mu_{2p}$, and $\mu_{\gamma }$, and variance $\sigma_{1p}^{2}$, $\sigma_{2p}^{2}$, and $\sigma_{\gamma }^{2}$ separately.*

For simplifying later calculation, we provide the following Lemma 2,

**Lemma** **2.** *[30]: If Z∼N(μ,σ), and w, A, B, D∈R, C∈R+, then the following holds:*

(13) $$\begin{array}{c} E_{Z}\left[\left(A-Z\right)\cdot \exp\left(-{\left(B-Z\right)}^{2}/2C\right)\right]\\ =\int_{-\infty }^{+\infty }\left(A-Z\right)\cdot \exp\left(-{\left(B-Z\right)}^{2}/2C\right)\frac{1}{\sqrt{2\pi \sigma^{2}}}\exp\left[\frac{{\left(A-\mu \right)}^{2}}{2\sigma^{2}}\right]dA\\ =\sqrt{\frac{C}{\sigma^{2}+C}}\left[A-\frac{\sigma^{2}B+\mu C}{\sigma^{2}+C}\right]\exp\left[-\frac{{\left(B-\mu \right)}^{2}}{2\left(\sigma^{2}+C\right)}\right] \end{array}$$

Similar to the derivation of the two-phase model with deterministic parameters, we try to conduct the lifetime distribution with three cases, i.e., 0≤T<τ, T=τ, and T>τ.

Based on the property of Lemma 1, the analytical form of lifetime at 0≤T<τ can be calculated as shown in following equation,

(14) $$\begin{array}{c} f_{T}\left(t\right)=\frac{\xi -x_{0}}{\sqrt{2\pi t^{2}\left(t\sigma_{1}^{2}+t^{2}\sigma_{1p}^{2}\right)}}\exp\left[-\frac{{\left(\xi -x_{0}-\mu_{1p}t\right)}^{2}}{2(t\sigma_{1}^{2}+t^{2}\sigma_{1p}^{2})}\right],\;\;\;0<t\leq \tau \end{array}$$

**Proof.**  See Appendix B. □

As discussed before, in order to derive the lifetime distribution at T=τ and T>τ, we should first obtain the expression of $x_{\tau^{-}}$ under the concept of FPT. It is noted that the PDF of of $x_{\tau^{-}}$ will be changed due to the randomness of the first phase model. Owing to $\mu_{1}∼N\left(\mu_{1p},\sigma_{1p}\right)$ and the property of Gaussian distribution, we can obtain the following result,

(15) $$\begin{array}{c} g_{\tau^{-}}\left(x_{\tau^{-}}|\mu_{1p},\sigma_{1p}\right)=\left[1-\exp\left(-\frac{4\xi^{2}-4x_{\tau^{-}}\xi }{2\sigma_{1}^{2}\tau }\right)\right]\frac{1}{\sqrt{2\pi (\tau \sigma_{1}^{2}+\tau^{2}\sigma_{1p}^{2})}}\exp\left[-\frac{{\left(x_{\tau^{-}}-\mu_{1p}\tau \right)}^{2}}{2(\tau \sigma_{1}^{2}+\tau^{2}\sigma_{1p}^{2})}\right]\\ =\frac{\exp\left[-\frac{{\left(x_{\tau^{-}}-\mu_{1p}\tau \right)}^{2}}{2(\tau \sigma_{1}^{2}+\tau^{2}\sigma_{1p}^{2})}\right]-\exp\left(\frac{2\mu_{1p}\xi }{\sigma_{1}^{2}}\;+\;\frac{2(\xi^{2}\sigma_{1p}^{4}\tau \;+\;\xi^{2}\sigma_{1p}^{2}\sigma_{1}^{2})}{\left(\sigma_{1}^{2}\;+\;\tau \sigma_{1p}^{2}\right)\sigma_{1}^{4}}\right)\exp\left[-\frac{{\left(x_{\tau^{-}}\;-\;2\xi \;-\;\mu_{1p}\tau \;-\;\frac{2\xi \sigma_{1p}^{2}\tau }{\sigma_{1}^{2}}\right)}^{2}}{2(\tau \sigma_{1}^{2}\;+\;\tau^{2}\sigma_{1p}^{2})}\right]}{\sqrt{2\pi (\tau \sigma_{1}^{2}+\tau^{2}\sigma_{1p}^{2})}} \end{array}$$

Next, we can obtain the PDF of $x_{\tau }$ based on $x_{\tau }=x_{\tau^{-}1}+\gamma$ as follows,

(16) $$\begin{array}{cc} g_{\tau }\left(x_{\tau }\right)= & \sqrt{\frac{1}{2\pi \left(\sigma_{a}^{2}+\sigma_{b}^{2}\right)}}\exp\left(-\frac{{\left(\mu_{a}-\mu_{b}\right)}^{2}}{2\left(\sigma_{a}^{2}+\sigma_{b}^{2}\right)}\right)\left[1-\Phi \left(-\frac{\xi \left(\sigma_{a}^{2}+\sigma_{b}^{2}\right)-\mu_{b}\sigma_{a}^{2}-\mu_{a}\sigma_{b}^{2}}{\sqrt{\sigma_{a}^{2}\sigma_{b}^{2}\left(\sigma_{a}^{2}+\sigma_{b}^{2}\right)}}\right)\right]\\ & -\exp\left(\frac{2\mu_{1p}\xi }{\sigma_{1}^{2}}+\frac{2\xi^{2}\sigma_{1p}^{2}}{\sigma_{1}^{4}}\right)\sqrt{\frac{1}{2\pi \left(\sigma_{a}^{2}+\sigma_{c}^{2}\right)}}\exp\left(-\frac{{\left(\mu_{a}-\mu_{c}\right)}^{2}}{2\left(\sigma_{a}^{2}+\sigma_{c}^{2}\right)}\right)\\ & \times \left[1-\Phi \left(-\frac{\xi \left(\sigma_{a}^{2}+\sigma_{c}^{2}\right)-\mu_{c}\sigma_{a}^{2}-\mu_{a}\sigma_{c}^{2}}{\sqrt{\sigma_{a}^{2}\sigma_{c}^{2}\left(\sigma_{a}^{2}+\sigma_{c}^{2}\right)}}\right)\right] \end{array}$$

where

(17) $$\begin{array}{c} \begin{array}{c} \mu_{a}=x_{\tau }-\mu_{\gamma },\;\mu_{b}=\mu_{1p}\tau_{1},\;\mu_{c}=\mu_{1p}\tau_{1}+2\xi +\frac{\sigma_{1p}^{2}\tau }{\sigma_{1}^{2}}\\ \sigma_{a}=\sigma_{\gamma },\;\sigma_{b}=\sigma_{c}=\sqrt{\tau_{1}\sigma_{1}^{2}+\tau_{1}^{2}\sigma_{1p}^{2}},\; \end{array} \end{array}$$

**Proof.**  See Appendix C. □

In this way, we can obtain the lifetime distribution at T=τ and T>τ separately based on the law of total probability.

(18) $$\begin{array}{c} \mathrm{Pr}\left(T=\tau \right)=\mathrm{Pr}\left(x_{\tau }=x_{\tau^{-}}+\gamma \geq \xi \right)=\int_{\xi }^{+\infty }g_{\tau }\left(x_{\tau }\right)dx_{\tau } \end{array}$$

and,

(19) $$\begin{array}{c} f_{T}\left(t\right)=\int_{-\infty }^{\xi }\frac{\xi -x_{\tau }}{\sqrt{2\pi \sigma_{2}^{2}{\left(t-\tau \right)}^{3}}}\exp\left[-\frac{{\left(\xi -x_{\tau }-\mu_{2}\left(t-\tau \right)\right)}^{2}}{2\sigma_{2}^{2}\left(t-\tau \right)}\right]g_{\tau }\left(x_{\tau }\right)dx_{\tau },\;\;T>\tau \end{array}$$

However, it is worth mentioning that the analytical expression of the above integrals is difficult to solve due to the complex form in (16), (18), and (19). Fortunately, there is only univariate integral, and then (19) can be solve by some well-developed numerical calculation methods. In addition, if the time of the change point is not given in advance, a common way is to define it as a random variable. In this case, the lifetime’s distribution can be rewritten as,

(20) $$\begin{array}{c} f_{T}\left(t\right)=\int_{0}^{+\infty }f_{T}\left(t|\tau \right)p\left(\tau \right)d\tau \end{array}$$

where p(τ) is the PDF of the changing time.

### 3.3. Lifetime Estimation for Multi-Phase Degradation Model

As to the multi-phase model, we should classify its lifetime into several parts according to the number of its degradation phase. It is assumed that the degradation model is defined as shown in (2). Then, we firstly consider a simplest case i.e., lifetime belong to 0<T<τ, the lifetime distribution is just determined by the first phase and holds the following form,

(21) $$\begin{array}{c} f_{T}\left(t\right)=\frac{\xi -x_{0}}{\sqrt{2\pi \sigma_{1}^{2}t^{3}}}\exp\left[-\frac{{\left(\xi -x_{0}-\mu_{1}t\right)}^{2}}{2\sigma_{1}^{2}t}\right],0<T<\tau_{1} \end{array}$$

Similar to the two-phase model, the key to lifetime estimation is how to formulate the PDF of $x_{\tau_{i}^{-}}$. Under this consideration, we first try to build the relationship between $x_{\tau_{i-1}^{-}}$ and $x_{\tau_{i}^{-}}$. Based on the result in Section 3.1, we can attain the following result,

(22) $$\begin{array}{c} \begin{array}{c} g_{\tau_{i}^{-}}\left(x_{\tau_{i}^{-}}|x_{\tau_{i-1}}\right)=\frac{1}{\sqrt{2\pi \Delta \tau_{i}\sigma_{i}^{2}}}\left\{\exp\left[-\frac{{\left(x_{\tau_{i}^{-}}-x_{\tau_{i-1}}-\mu_{i}\Delta \tau_{i}\right)}^{2}}{2\sigma_{i}^{2}\Delta \tau_{i}}\right]\right.\\ \left.-\exp\left(\frac{2\mu_{i}\left(\xi -x_{\tau_{i-1}}\right)}{\sigma_{i}^{2}}\right)\exp\left[-\frac{{\left(x_{\tau_{i}^{-}}+x_{\tau_{i-1}}-2\xi -\mu_{i}\Delta \tau_{i}\right)}^{2}}{2\sigma_{i}^{2}\Delta \tau_{i}}\right]\right\} \end{array} \end{array}$$

where $\Delta \tau_{i}\;=\;\tau_{i}-\tau_{i-1}$. Then, according to $x_{\tau_{i-1}}\;=\;x_{\tau_{i-1}^{-}}+\gamma_{i}$, we can further obtain,

(23) $$\begin{array}{c} \begin{array}{c} g_{\tau_{i}^{-}}\left(x_{\tau_{i}^{-}}|x_{\tau_{i-1}^{-}}\right)=\frac{1}{\sqrt{2\pi \Delta \tau_{i}\sigma_{i}^{2}}}\left\{\exp\left[-\frac{{\left(x_{\tau_{i}^{-}}-x_{\tau_{i-1}^{-}}+\gamma_{i}-\mu_{i}\Delta \tau_{i}\right)}^{2}}{2\sigma_{i}^{2}\Delta \tau_{i}}\right]\right.\\ \left.-\exp\left(\frac{2\mu_{i}\left(\xi -x_{\tau_{i-1}^{-}}-\gamma_{i}\right)}{\sigma_{i}^{2}}\right)\exp\left[-\frac{{\left(x_{\tau_{i}^{-}}+x_{\tau_{i-1}^{-}}-\gamma_{i}-2\xi -\mu_{i}\Delta \tau_{i}\right)}^{2}}{2\sigma_{i}^{2}\Delta \tau_{i}}\right]\right\} \end{array} \end{array}$$

In this way, we can further obtain $g_{\tau_{i}^{-}}\left(x_{\tau_{i}^{-}}\right)$ with a multi-integral form as follows,

(24) $$\begin{array}{c} \begin{array}{c} g_{\tau_{i}^{-}}\left(x_{\tau_{i}^{-}}\right)=\int_{-\infty }^{{\tilde{\xi }}_{i-1}}\ldots \int_{-\infty }^{{\tilde{\xi }}_{2}}\int_{-\infty }^{{\tilde{\xi }}_{1}}g_{\tau_{1}}\left(x_{\tau_{1}^{-}}\right)g_{\tau_{2}}\left(x_{\tau_{2}^{-}}|x_{\tau_{1}^{-}}\right)g_{\tau_{3}}\left(x_{\tau_{3}^{-}}|x_{\tau_{2}^{-}}\right)\ldots g_{\tau_{i}}\left(x_{\tau_{i}^{-}}|x_{\tau_{i-1}^{-}}\right)dx_{\tau_{1}^{-}}dx_{\tau_{2}^{-}}\ldots dx_{\tau_{i-1}^{-}} \end{array} \end{array}$$

where ${\tilde{\xi }}_{i-1}=\xi -I\left(\gamma_{i}\leq 0\right)\cdot \gamma_{i}$. In this way, the multi-integral expression of $g_{\tau_{i}^{-}}\left(x_{\tau_{i}^{-}}\right)$ has been attained, and then the distribution of the lifetime at $T\;=\;\tau_{i}$ and $\tau_{i-1}<T<\tau_{i}$ can be calculated similar to the method of two-phase model as shown in (18) and (19),

(25) $$\begin{array}{c} \begin{array}{c} \mathrm{Pr}\left(T=\tau_{i}\right)=\mathrm{Pr}\left(x_{\tau_{i}}=x_{\tau_{i}^{-}}+\gamma_{i}\geq \xi_{i}\right)=I\left(\gamma \leq 0\right)\cdot 0+I\left(\gamma >0\right)\left[\int_{\xi -\gamma_{i}}^{\xi }g_{\tau_{i}^{-}}\left(x_{\tau_{i}^{-}}\right)dx_{\tau_{i}^{-}}\right] \end{array} \end{array}$$

and,

(26) $$\begin{array}{c} f_{T}\left(t\right)=\int_{-\infty }^{{\tilde{\xi }}_{i-1}}\frac{\xi -x_{\tau_{i-1}}}{\sqrt{2\pi \sigma_{i}^{2}t^{3}}}\exp\left[-\frac{{\left(\xi -x_{\tau_{i-1}}-\mu_{i}t\right)}^{2}}{2\sigma_{i}^{2}\left(t-\tau_{i-1}\right)}\right]dx_{\tau_{i-1}},\;\;\tau_{i-1}<t<\tau_{i} \end{array}$$

It is noted that the time of the change point is known in advance and it is defined as a fixed value in this subsection. If the changing time is random, we can utilize the law of the total probability similar to (20).

**Remark** **2.** *For RUL estimation, RUL can be regard as the lifetime by letting the initial time $t_{0}=t_{k}$ according to the definition as shown in (3) and (4). Thus, RUL can be derived directly based on the relationship between lifetime and RUL. Take the two-phase model as an example, if the change point does not appear, i.e., $\tau >t_{k}$, we can estimate the RUL based on the results of lifetime estimation by letting $t_{0}=t_{k}$, $x_{0}=x_{k}$, and $\tau =\tau -t_{k}$. On the other hand, if the change point has appeared, the degradation process become single-phase model, and RUL can be easily obtained by traditional way. Due to the limited space, the detailed expression of RUL estimation is omitted in this paper.*

## 4. Parameter Identification

In this section, to the implementation of our method, we take the two-phase mode with random effect for example.

### 4.1. Off-Line Method

Firstly, we assume that there are *n* degradation devices from the same batch. That is to say, there are *n* sets of degradation data separately, i.e., $X=\{X_{1},X_{2},\ldots ,X_{n}\}$. In addition, we further define that $X_{i}=\left\{x_{i,0},x_{i,1},\ldots ,x_{i,m_{i}}\right\}$ denotes the observation of the *i*-th device at time $\left\{t_{i,0},t_{i,1},\ldots ,t_{i,m_{i}}\right\}$. For simplicity, we only consider the case that the sampling interval is fixed i.e., $\Delta t=t_{i,j}-t_{i,j-1}$ in this paper.

Then, based on the property of Wiener process the likelihood function of $X_{i}$ can be formulated as follows,

(27) $$\begin{array}{c} \ln L\left(\mu_{i},\sigma_{i},{\tilde{\tau }}_{i},\gamma_{i}|X_{i}\right)=\sum_{j=1}^{{\tilde{\tau }}_{i}}\ln\frac{1}{\sqrt{2\pi \sigma_{1}^{2}\Delta t}}\exp\left[\frac{{\left(x_{i,j}-x_{i,j-1}-\mu_{1,i}\Delta t\right)}^{2}}{2\sigma_{1}^{2}\Delta t}\right]\\ +\sum_{j={\tilde{\tau }}_{i}+2}^{m_{i}}\ln\frac{1}{\sqrt{2\pi \sigma_{2}^{2}\Delta t}}\exp\left[\frac{{\left(x_{i,j}-x_{i,j-1}-\mu_{2,i}\Delta t\right)}^{2}}{2\sigma_{2}^{2}\Delta t}\right]\\ +\ln\frac{1}{\sqrt{2\pi \left[\sigma_{1}^{2}\left(\tau_{i}-{\tilde{\tau }}_{i}\Delta t\right)+\sigma_{2}^{2}\left({\tilde{\tau }}_{i}\Delta t+\Delta t-\tau_{i}\right)\right]}}\exp\left[\frac{{\left(x_{i,{\tilde{\tau }}_{i}+1}-x_{i,{\tilde{\tau }}_{i}}-\gamma_{i}-\mu_{1,i}\left(\tau_{i}-{\tilde{\tau }}_{i}\Delta t\right)-\mu_{2,i}\left({\tilde{\tau }}_{i}\Delta t+\Delta t-\tau_{i}\right)\right)}^{2}}{2\sigma_{1}^{2}\left(\tau_{i}-{\tilde{\tau }}_{i}\Delta t\right)+2\sigma_{2}^{2}\left({\tilde{\tau }}_{i}\Delta t+\Delta t-\tau_{i}\right)}\right] \end{array}$$

where ${\tilde{\tau }}_{i}=\left\lfloor \tau_{i}/\Delta t\right\rfloor$, where ⌊⌋ is the round down operator, and $\gamma_{i}$, $\mu_{1,i}$, $\sigma_{1,i}$, $\mu_{2,i}$, and $\sigma_{2,i}$ denote the parameters of the two-phase model for the *i*-th device, and $\tau_{i}$ is the changing time, i.e., ${\tilde{\tau }}_{i}\in 0,1,2,\ldots ,m_{i}$. So $\left\{x_{i,0},x_{i,1},\ldots ,x_{i,{\tilde{\tau }}_{i}}\right\}$ is the observation at first phase, and $\left\{x_{i,{\tilde{\tau }}_{i}+1},x_{i,{\tilde{\tau }}_{i}+2},\ldots ,x_{i,m_{i}}\right\}$ is the observation at the second phase.

It is found that the ${\hat{\mu }}_{1,i}$ and ${\hat{\mu }}_{2,i}$ can be seen as the observation of the random variables $\mu_{1}$ and $\mu_{2}$. If the changing time is known, it is natural to regard $\mu_{1,i}$, and $\mu_{2,i}$ as the latent variables and then the EM algorithm is adopted for calculation. According to the EM algorithm, the completed likelihood can be formulated as follows,

(28) $$\begin{array}{c} \ln L\left(\Xi \left|X,Z\right.\right)=\ln\prod_{i=1}^{n}p(X_{i},Z_{i}|\Xi )=\sum_{i=1}^{n}\ln\left(p\left(Z_{i}|\Xi \right)p\left(X_{i}|Z_{i},\Xi \right)\right) \end{array}$$

where $Z_{i}=\left\{\mu_{1,i},\mu_{2,i}\right\}$ denotes the latent variables and $\Xi =\{\sigma_{1},\sigma_{2},\mu_{1p},\sigma_{1p},\mu_{2p},\sigma_{2p}\}$ represents all parameters of the degradation model.

Let ${\hat{\Xi }}^{\left(k\right)}=\left\{{\hat{\sigma }}_{1}^{\left(k\right)},{\hat{\sigma }}_{2}^{\left(k\right)},{\hat{\mu }}_{1p}^{\left(k\right)},{\hat{\sigma }}_{1p}^{\left(k\right)},{\hat{\mu }}_{2p}^{\left(k\right)},{\hat{\sigma }}_{2p}^{\left(k\right)}\right\}$ denote estimates in the *k*-th step, and then we can obtain the next iteration ${\hat{\Xi }}^{\left(k+1\right)}$ as follows according to the EM algorithm.

(29) $$\begin{array}{cc} {\hat{\mu }}_{1p}^{\left(k+1\right)}= & \frac{1}{n}\sum_{i=1}^{n}\frac{\left(x_{i,{\tilde{\tau }}_{i}}-x_{i,0}\right)\Delta t\sigma_{1p}^{2,(k)}+\sigma_{1,i}^{2,(k)}\Delta t\mu_{1p}^{\left(k\right)}}{{\tilde{\tau }}_{i}\sigma_{1p}^{2,(k)}\Delta t^{2}+\sigma_{1,i}^{2,(k)}\Delta t}\\ {\hat{\sigma }}_{1p}^{\left(k+1\right)}= & \sqrt{\frac{1}{n}\sum_{i=1}^{n}\left(E\left[\mu_{1,i}^{2}|X_{i},{\hat{\Xi }}^{\left(k\right)}\right]-E^{2}\left[\mu_{1,i}|X_{i},{\hat{\Xi }}^{\left(k\right)}\right]\right)}\\ {\hat{\mu }}_{2p}^{\left(k+1\right)}= & \frac{1}{n}\sum_{i=1}^{n}\frac{\left(x_{i,m_{i}}-x_{i,{\tilde{\tau }}_{i}+1}\right)\Delta t\sigma_{2p}^{2,(k)}+\sigma_{2,i}^{2,(k)}\Delta t\mu_{2p}^{\left(k\right)}}{\left(m_{i}-{\tilde{\tau }}_{i}-1\right)\sigma_{2p}^{2,(k)}\Delta t^{2}+\sigma_{2,i}^{2,(k)}\Delta t}\\ {\hat{\sigma }}_{2p}^{\left(k+1\right)}= & \sqrt{\frac{1}{n}\sum_{i=1}^{n}\left(E\left[\mu_{2,i}^{2}|X_{i},{\hat{\Xi }}^{\left(k\right)}\right]-E^{2}\left[\mu_{2,i}|X_{i},{\hat{\Xi }}^{\left(k\right)}\right]\right)}\\ {\hat{\sigma }}_{1}^{\left(k+1\right)}= & \sqrt{\frac{\sum_{i=1}^{n}\left\{\sum_{j=1}^{{\tilde{\tau }}_{i}}{\left(x_{i,j}-x_{i,j-1}\right)}^{2}-2E\left[\mu_{1,i}|X_{i},{\hat{\Xi }}^{\left(k\right)}\right]\sum_{j=1}^{{\tilde{\tau }}_{i}}\left(x_{i,j}-x_{i,j-1}\right)+{\tilde{\tau }}_{i}E\left[\mu_{1,i}^{2}|X_{i},{\hat{\Xi }}^{\left(k\right)}\right]\right\}}{\sum_{i=1}^{n}{\tilde{\tau }}_{i}\Delta t}}\\ {\hat{\sigma }}_{2}^{\left(k+1\right)}= & \sqrt{\frac{\sum_{i=1}^{n}\left\{\sum_{j={\tilde{\tau }}_{i}+2}^{m_{i}}{\left(x_{i,j}-x_{i,j-1}\right)}^{2}-2E\left[\mu_{2,i}|X_{i},{\hat{\Xi }}^{\left(k\right)}\right]\sum_{j={\tilde{\tau }}_{i}+2}^{m_{i}}\left(x_{i,j}-x_{i,j-1}\right)+\left(m_{i}-{\tilde{\tau }}_{i}-1\right)E\left[\mu_{2,i}^{2}|X_{i},{\hat{\Xi }}^{\left(k\right)}\right]\right\}}{\sum_{i=1}^{n}\left(m_{i}-{\tilde{\tau }}_{i}-1\right)\Delta t}} \end{array}$$

where

(30) $$\begin{array}{cc} E[\mu_{1,i}|X_{i},{\hat{\Xi }}^{\left(k\right)}]= & \frac{\left(x_{i,{\tilde{\tau }}_{i}}-x_{i,0}\right)\Delta t\sigma_{1p}^{2,(k)}+\sigma_{1}^{2,(k)}\Delta t\mu_{1p}^{\left(k\right)}}{{\tilde{\tau }}_{i}\sigma_{1p}^{2,(k)}\Delta t^{2}+\sigma_{1}^{2,(k)}\Delta t}\\ E[\mu_{1,i}^{2}|X_{i},{\hat{\Xi }}^{\left(k\right)}]= & \frac{\sigma_{1p}^{2,(k)}\sigma_{1}^{2,(k)}\Delta t}{{\tilde{\tau }}_{i}\sigma_{1p}^{2,(k)}\Delta t^{2}+\sigma_{1}^{2,(k)}\Delta t}+{\left[\frac{\left(x_{i,{\tilde{\tau }}_{i}}-x_{i,0}\right)\Delta t\sigma_{1p}^{2,(k)}+\sigma_{1}^{2,(k)}\Delta t\mu_{1p}^{\left(k\right)}}{{\tilde{\tau }}_{i}\sigma_{1p}^{2,(k)}\Delta t^{2}+\sigma_{1}^{2,(k)}\Delta t}\right]}^{2}\\ E[\mu_{2,i}|X_{i},{\hat{\Xi }}^{\left(k\right)}]= & \frac{\left(x_{i,m_{i}}-x_{i,{\tilde{\tau }}_{i}+1}\right)\Delta t\sigma_{2p}^{2,(k)}+\sigma_{2}^{2,(k)}\Delta t\mu_{2p}^{\left(k\right)}}{\left(m_{i}-{\tilde{\tau }}_{i}-1\right)\sigma_{2p}^{2,(k)}\Delta t^{2}+\sigma_{2}^{2,(k)}\Delta t}\\ E[\mu_{2,i}^{2}|X_{i},{\hat{\Xi }}^{\left(k\right)}]= & \frac{\sigma_{2p}^{2,(k)}\sigma_{2}^{2,(k)}\Delta t}{\left(m_{i}-{\tilde{\tau }}_{i}-1\right)\sigma_{2p}^{2,(k)}\Delta t^{2}+\sigma_{2}^{2,(k)}\Delta t}+{\left[\frac{\left(x_{i,m_{i}}-x_{i,{\tilde{\tau }}_{i}+1}\right)\Delta t\sigma_{2p}^{2,(k)}+\sigma_{2}^{2,(k)}\Delta t\mu_{2p}^{\left(k\right)}}{\left(m_{i}-{\tilde{\tau }}_{i}-1\right)\sigma_{2p}^{2,(k)}\Delta t^{2}+\sigma_{2}^{2,(k)}\Delta t}\right]}^{2} \end{array}$$

**Proof.**  See Appendix D. □

It is noted that when the changing time is unknown the change point should be detected. The common way is to adopt the maximum likelihood estimation to obtain the estimates ${\hat{\tau }}_{i}$ and ${\hat{\gamma }}_{i}$ and then calculate the distribution of τ and γ according to these estimates, which can be found in our pervious work [25]. For space limitation, it is omitted in this paper.

### 4.2. On-Line Updating Method

In this subsection, we concentrate on how to update the parameters online for a certain operating equipment by using newly arriving degradation data, where the results in the off-line part are regarded as the prior information. If the current time is $t_{\kappa }$, we can obtain the degradation data $X_{0:\kappa }=\left\{x_{0},x_{1},\ldots ,x_{\kappa }\right\}$. It is worth mentioning that when the change point does not appear (i.e., $t_{\kappa }\leq \tau$) only the parameters at the first phase should be updated. On the contrary, if $t_{\kappa }>\tau$, we just update the parameters at the second phase.

Let $\mu_{1p,0}$, $\sigma_{1p,0}$, $\mu_{2p,0}$, and $\sigma_{2p,0}$ denote the prior value of $\mu_{1p}$, $\sigma_{1p}$, $\mu_{2p}$, and $\sigma_{2p}$. As discussed before, when $t_{\kappa }\leq \tau$, all observations $X_{0:k}=\left\{x_{0},x_{1},\ldots ,x_{\kappa }\right\}$ could be used for updating. According to the Bayesian rule, we can have the following results,

(31) $$\begin{array}{c} p\left(\mu_{1}|X_{0:\kappa }\right)\propto p\left(X_{0:\kappa }|\mu_{1}\right)p\left(\mu_{1}\right) \end{array}$$

where

(32) $$\begin{array}{cc} p(X_{0:\kappa }|\mu_{1})= & \prod_{i=1}^{\kappa }\frac{1}{\sqrt{2\pi \sigma_{1}^{2}\Delta t}}\exp\left[-\frac{{\left(x_{i}-x_{i-1}-\mu_{1}\Delta t\right)}^{2}}{2\sigma_{1}^{2}\Delta t}\right]\\ p(\mu_{1})= & \frac{1}{\sqrt{2\pi \sigma_{1p,0}^{2}}}\exp\left[-\frac{{\left(\mu_{1}-\mu_{1p,0}\right)}^{2}}{2\sigma_{1p,0}^{2}}\right] \end{array}$$

Since $p(X_{0:\kappa }|\mu_{1})$ and $p(\mu_{1})$ follow Gaussian distribution, then we can further attain the posterior distribution,

(33) $$\begin{array}{c} p\left(\mu_{1}|X_{0:\kappa }\right)=\frac{1}{\sqrt{2\pi \sigma_{1p}^{2}}}\exp\left[-\frac{{\left(\mu_{1}-\mu_{1p}\right)}^{2}}{2\sigma_{1p}^{2}}\right] \end{array}$$

with

(34) $$\begin{array}{c} \mu_{1p}=\frac{\mu_{1p,0}\sigma_{1}^{2}+\left(x_{\kappa }-x_{0}\right)\sigma_{1p,0}^{2}}{\left(t_{\kappa }-t_{0}\right)\sigma_{1p,0}^{2}+\sigma_{1}^{2}},\;\;\;\sigma_{1p}=\sqrt{\frac{\sigma_{1}^{2}\sigma_{1p,0}^{2}}{\left(t_{\kappa }-t_{0}\right)\sigma_{1p,0}^{2}+\sigma_{1}^{2}}} \end{array}$$

On the other hand, when $t_{\kappa }>\tau$, we will update the posterior distribution of $\mu_{2}$ by using the observation $X_{0:k}=\left\{x_{\tau +1},x_{\tau +2},\ldots ,x_{\kappa }\right\}$.

(35) $$\begin{array}{c} p\left(\mu_{2}|X_{0:\kappa }\right)=\frac{1}{\sqrt{2\pi \sigma_{2p}^{2}}}\exp\left[-\frac{{\left(\mu_{2}-\mu_{2p}\right)}^{2}}{2\sigma_{2p}^{2}}\right] \end{array}$$

with

(36) $$\begin{array}{c} \mu_{2p}=\frac{\mu_{2p,0}\sigma_{2}^{2}+\left(x_{\kappa }-x_{\tilde{\tau }+1}\right)\sigma_{2p,0}^{2}}{\left(t_{\kappa }-t_{\tilde{\tau }+1}\right)\sigma_{2p,0}^{2}+\sigma_{2}^{2}},\;\;\;\sigma_{2p}=\sqrt{\frac{\sigma_{2}^{2}\sigma_{2p,0}^{2}}{\left(t_{\kappa }-t_{\tilde{\tau }+1}\right)\sigma_{2p,0}^{2}+\sigma_{2}^{2}}} \end{array}$$

In this way, the estimates of $\mu_{1p}$, $\sigma_{1p}$, $\mu_{2p}$, and $\sigma_{2p}$ could be updated based on Bayesian rule. In addition, if the change point is not given in advance, the change point detection method is the same with the off-line approach, which is omitted in this subsection.

## 5. Case Study

In this section, two numerical examples are provided: (1) A numerical example is given to verify the proposed approach; (2) A practical example of a gyro is provided for illustration.

### 5.1. Numerical Case

In this subsection, we attempt to verify the result of our method for lifetime estimation and parameter identification.

Firstly, we compare the result of lifetime estimation based on our method with the result of Monte Carlo (MC) method. In this paper, we consider four cases: fixed positive jump with a given changing time, fixed negative jump with a given changing time, random jump with a given changing time, and random jump with a random changing time. For the first case, the degradation model is defined as (5) and its parameters are given as: $\mu_{1}=0.4$, $\sigma_{1}=0.2$, $\mu_{2}=0.2$, $\sigma_{2}=0.1$, τ=10, γ=0.5, and ξ=4; For the second case, the model form and the parameters are the same with the first case expect γ=−0.5; For the third case, we further consider the degradation with random effect, where $\mu_{1}$, $\mu_{2}$, and γ follow Gaussian distribution with $\mu_{1p}=0.4$, $\mu_{2p}=0.2$, and $\mu_{\gamma }=0.5$, and $\sigma_{1p}=0.1$, $\sigma_{2p}=0.05$, and $\sigma_{\gamma }=1$; For the forth case, distinguished with the third case, the changing time τ is random, where we let 10×τ follow a gamma distribution with α=50 and β=2.

**Figure 2:**
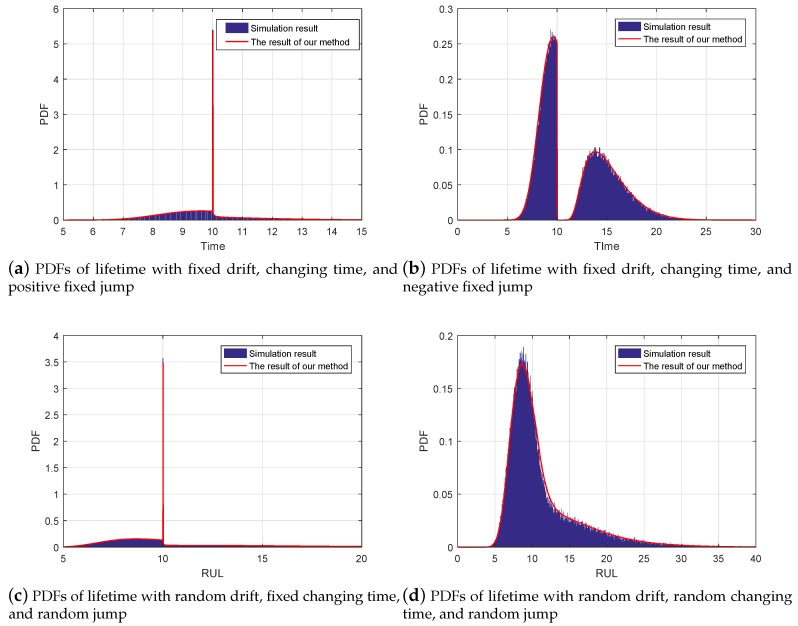
The estimated lifetime PDFs under different conditions.

For a better illustration, we adopt the MC method to generate the 1,000,000 sets of degradation paths and then collect their FPTs as the result of the lifetime, where the we set initial time and initial degradation value as $t_{0}=0$ and $x_{0}=0$ for simplicity. In this way, we can obtain the lifetime’s distribution of the given degradation model in a numerical way. Following Figure 2 shows that the comparison between the simulation results and our results. By this comparison, it it noted that our results can achieve accurate lifetime estimation, which can illustrate the effectiveness of our method. In these four cases, since neither of the drift and diffusion coefficients at two phases are the same, Kong’s method requiring the same diffusion coefficients at two phases cannot work. In this way, it can be concluded that these comparisons can verify our approach in theory. It is worth mentioning that some estimated bias is still existed due to the numerical integral, step size of simulation, and simulation times.

**Figure 3:**
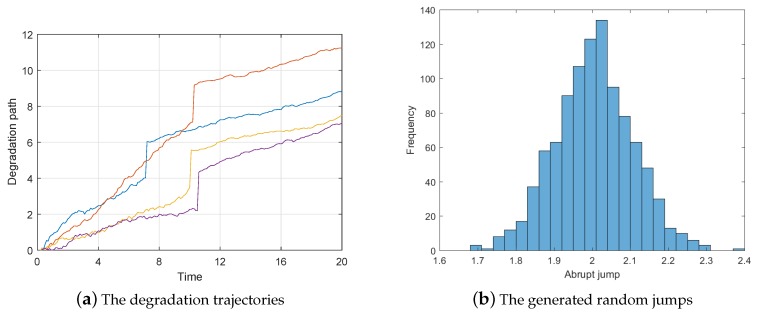
The examples of the simulation degradation process.

Next, we will introduce how to identify the degradation model based on our approach. For better illustration, we generate 50 degradation trajectories with random changing time, drift, and jump. Figure 3a shows several typical degradation paths of them, which exhibits obvious two-phase-jump feature similar to the practical cases in Figure 1. Figure 3b shows the statistical graph of the jump amplitude. Based on the proposed model identification method we can obtain the parameters’ estimation as shown in following Table 1,

From Table 1, we can find that the results of our method can approach the true value when more samples are adopted to identify model parameter. It can verify the effectiveness of our off-line model identification method. In this way, we have completed the off-line lifetime estimation and off-line model identification. Next, we will introduce how to do RUL predicting and parameters’ updating.

**Figure 4:**
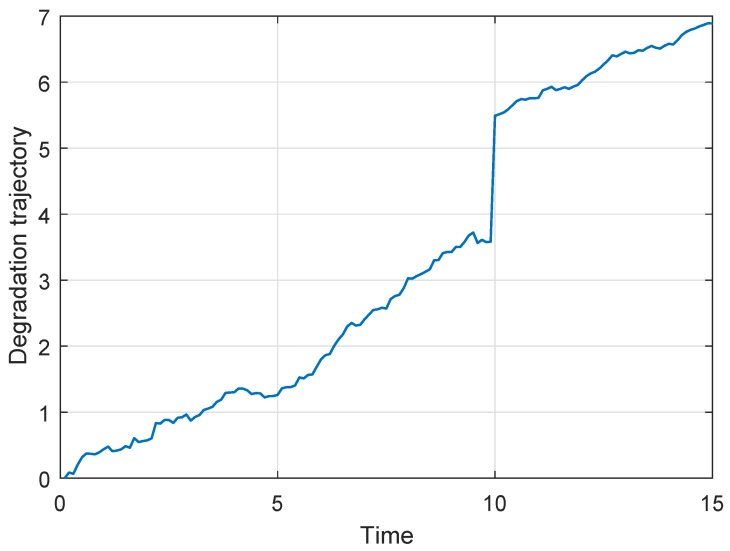
The single degradation process for illustration.

**Figure 5:**
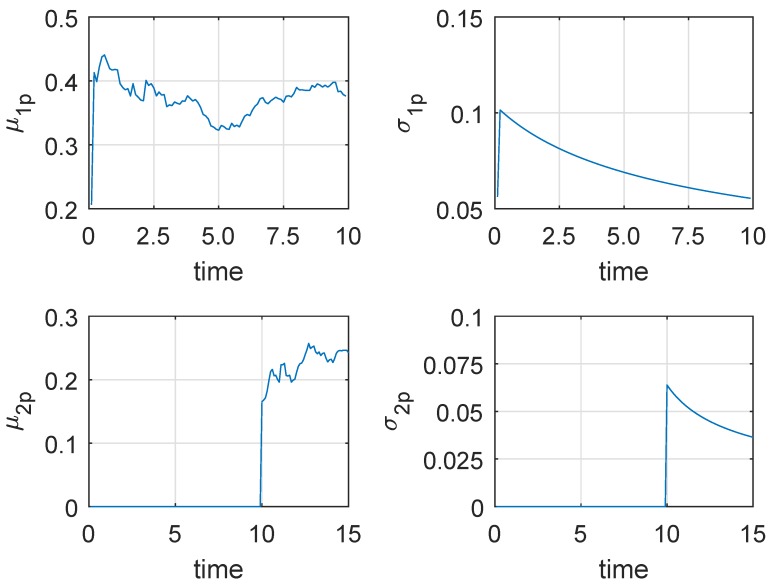
The updating of estimated parameters based on Bayesian rule.

Following Figure 4 shows a degradation process chosen from the 50 generated degradation paths for illustration, where the actual $\mu_{1}=0.36$ and $\mu_{2}=0.24$. Then if the new observed data are coming, the parameters’ estimates can be updated based on the method in Section 4.2. If the degradation process do not enter the second phase, only the parameters of the first phase model should be updated. Otherwise, we just need to update the parameters of the second phase model. In this way, we can obtain the parameters’ updating procedure as shown in following Figure 5, where the results of n = 10 samples are treated as the prior information. From Figure 5, we can find that $\sigma_{1p}$ and $\sigma_{2p}$ decrease gradually, which reflects that the uncertainty of the parameters’ estimates is reduced.

**Figure 6:**
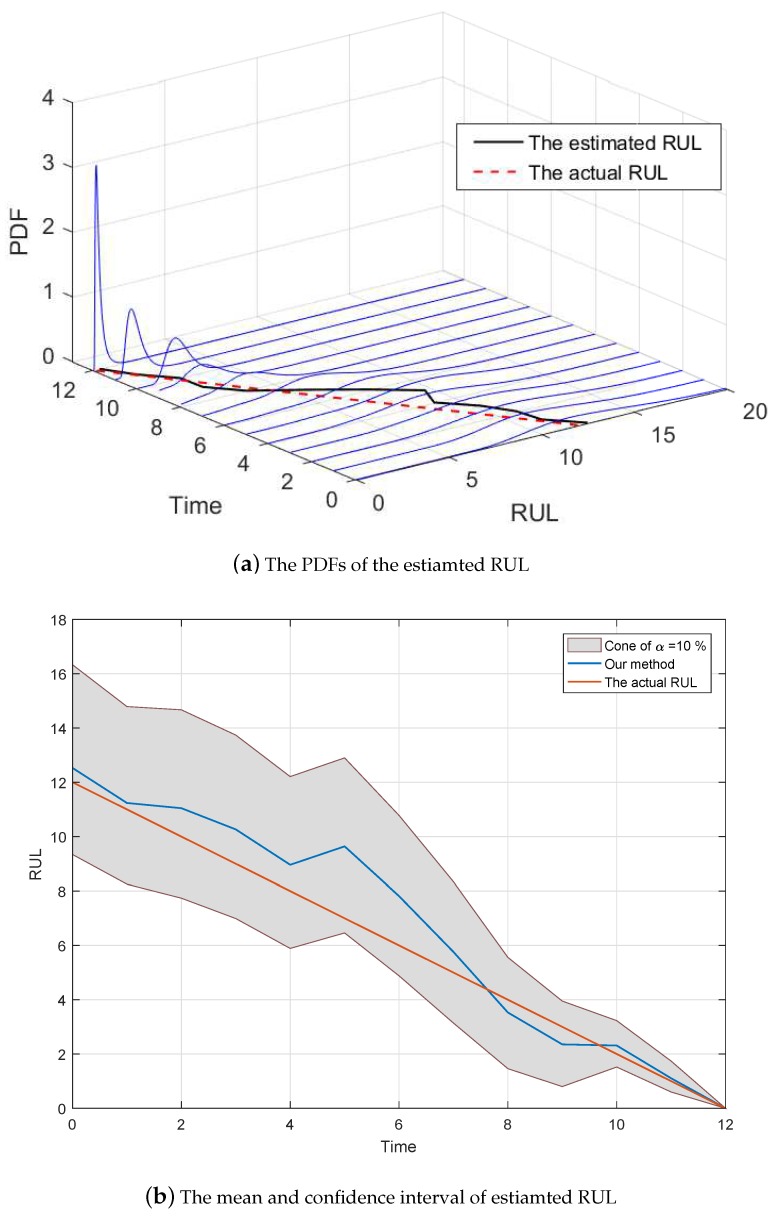
The comparison of the estimated RUL.

Since the size of observed data is not large enough, $\mu_{1p}$ and $\mu_{2p}$ do not converge to their actual value 0.36 and 0.24, and $\sigma_{1p}$ and $\sigma_{2p}$ do not approach 0 completely. Moreover, the estimated changing time is 95 rather than the real changing time 98. These may lead to some estimated bias of RUL estimation. Figure 6a shows the predicted RUL result, where the threshold ξ=6 and the actual lifetime is 12. Inspired by Saxena’s method [31], we further compare the mean and the confidence interval of the estimated RUL with the actual RUL.

From the numerical example, it could be concluded that our method cannot only reflect the degradation trajectory but also achieve more accurate estimated result, which could illustrate the advantage and effectiveness of our approach.

**Remark** **3.** *It is noteworthy that because the degradation data in this subsection are generated by numerical simulation, and Figure 2, Figure 3, Figure 4, Figure 5 and Figure 6 show the results of the numerically simulated data. Therefore, the units of these figures are omitted in this subsection.*

### 5.2. Practical Case

In this subsection, we continue to utilize the practical case of gyro to illustration our method. To better illustrate the application of our approach, we first introduce the following procedure to show how to realize the RUL estimation for the degradation device as shown in Table 2.

**Figure 7:**
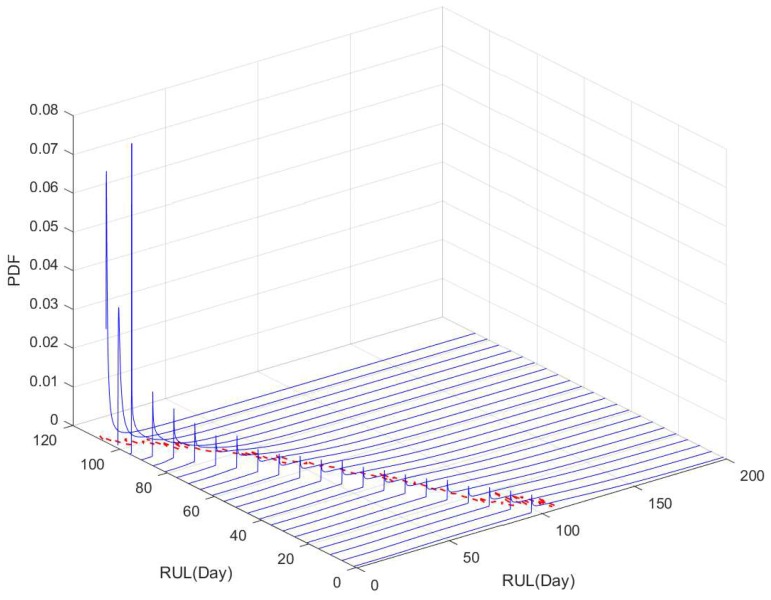
The PDFs of the estimated RUL based on our method.

According to the implementation procedures, we first use the two sets (i.e., blue line and yellow line in Figure 1) for off-line training based on the method in Section 4.1, and then treat these results as the prior information. Then we adopt the degradation processes of the other gyro (i.e., red line in Figure 1) to illustrate RUL estimation. We update the parameters’ estimates based on the Bayesian rule, and further obtain the estimated RUL’s distribution as shown in following Figure 7.

**Figure 8:**
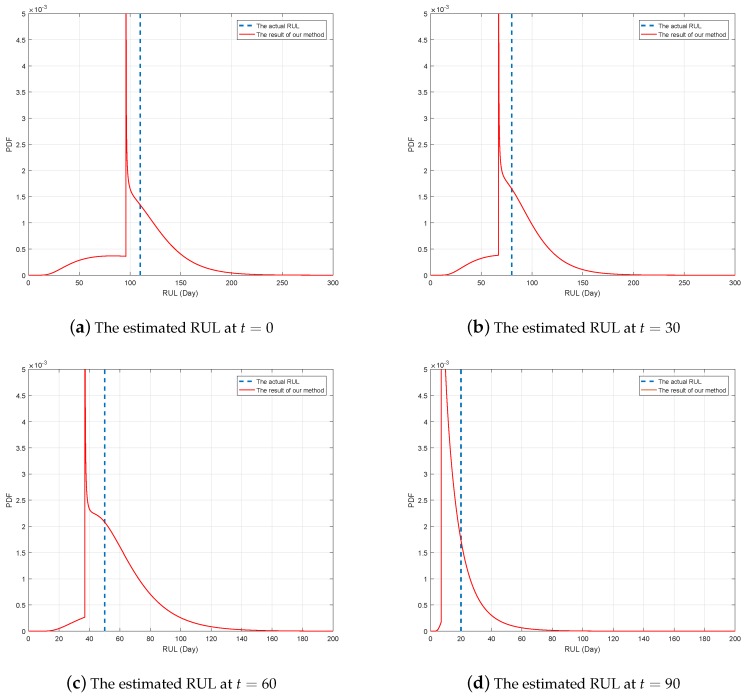
The estimated RUL based on our method at different time.

**Figure 9:**
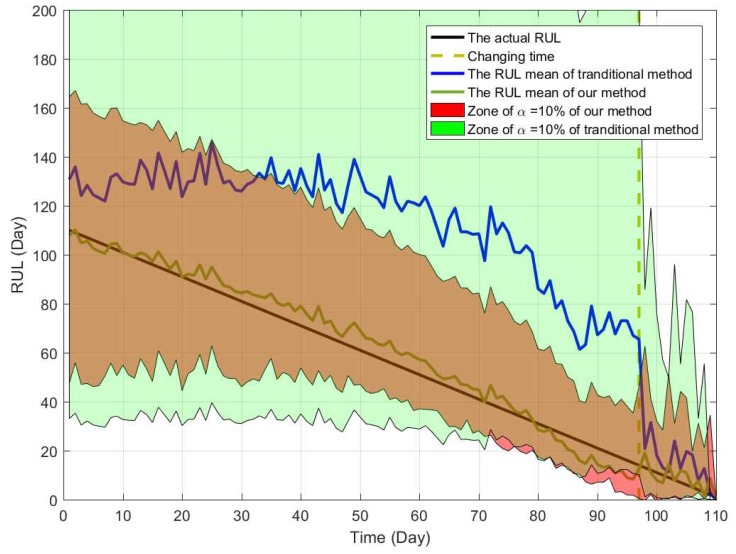
The comparison of estimated RUL between traditional method and our method.

In Figure 7, the black line denotes the actual RUL, and the blue lines reflect PDFs of estimated RUL at several different time. It should be noted that the PDF is not continuous at discussed before, and the failure probability at the change point is not shown in Figure 7. For a better illustration, Figure 8 shows the PDFs of the estimated RUL at four given time. We can find that the actual RUL has been included in the range of the estimated RUL’s PDF. Furthermore, we compare the obtained results based on our method with the traditional way. Following Figure 9 show the mean and confidence interval of the estimated RUL based on two methods, including our method and the traditional method of [30].

**Remark** **4.** *It is interesting to see that the distribution of RUL looks unnatural in Figure 8, which is mainly due to the change of degradation rate and the random jump. As discussion in Remark 2, the form of RUL’s distribution is similar to the lifetime’s when the change point does not appear. In our example, since the change time is τ=97, the lifetime’ distribution is not continuous and looks unnatural until τ≥97.*

From the comparison, it is can be concluded that our method can model the two-phase degradation process with random jump well and obtain the accurate result of the RUL estimation. In the traditional way, the estimated bias is much large duo to the effect of the random jump and the multi-phase pattern. That is to say, if we ignore the influence of the abrupt jump and the multi-phase pattern, the estimated bias cannot be avoided, which may cause inaccurate RUL estimation and improper maintenance arrangement. By comparison, our method can overcome this problem and obtain the more accurate result. Besides, it is noteworthy that since the degradation feature of three gyros are much different from each other, some estimated bias of RUL cannot be eliminated at all.

## 6. Conclusions

In this paper, we mainly concentrate on how to model the multi-phase degradation process with random jumps and estimate its lifetime. We first propose a multi-phase degradation model with abrupt jumps to describe this kind of degradation trajectories. Unlike existing work, we take a full account of the uncertainties of the first phase degradation process and the abrupt jump. Then we provide the method of lifetime estimation under the concept of FPT. In addition, an on-line and off-line parameters’ identification methods are proposed for facilitating practical usage. Finally, a numerical case and practical case are provided to illustrate our method.

However, although some effective results have been obtained in this paper, some challenging problems are still needed to investigate in future: (1) How to determine the number of degradation stage for some degradation process without obvious mechanism information; (2) How to derive the analytical expression of the lifetime (or RUL) for mutli-phase nonlinear degradation process with random jumps.

## Figures and Tables

**Table 1:** The parameters estimation with different sample size.

Sample Size	$\mu_{1p}$	$\sigma_{1p}$	$\sigma_{1}$	$\mu_{2p}$	$\sigma_{2p}$	$\sigma_{2}$	$\mu_{\gamma }$	$\sigma_{\gamma }$	α	β
n = 5	0.379	0.095	0.199	0.211	0.0403	0.0098	2.0241	0.0776	27.258	3.6466
n = 10	0.420	0.113	0.200	0.189	0.0371	0.0101	2.0330	0.0641	32.312	3.0722
n = 50	0.395	0.104	0.200	0.192	0.0474	0.0102	2.0169	0.0931	39.090	2.6001
True value	0.400	0.100	0.200	0.200	0.0500	0.0100	2.0000	0.1000	50.000	2.0000

**Table 2:** The implementation procedures of RUL estimation for the degradation device.

Algorithm Procedure:	
*Step 1.*	Identify the parameters by the historical data based on the method in Section 4.1.
*Step 2.*	Collect the operating degradation data, and then detect the appearing of the change point if the change time is not known.
*Step 3.*	Update the parameters of the first phase model based on the method in Section 4.2 until the the change point appears. Otherwise, update the parameters of the second phase model.
*Step 4.*	Estimate the RUL online based on the result in Section 3 and Remark 2.
*Step 5.*	Collect latest degradation data and then go to step 2 until degradation reaches the predefined failure threshold.

## Abbreviations

The following abbreviations are used in this manuscript:

RUL	Remaining useful life
FPT	First passage time
MLE	Maximum Likelihood Estimation
EM	Expectation maximum
PHM	Prognostic and health management
FPT	First passage time
PDF	Probability density function
CDF	Cumulative distribution function
MC	Monte Carlo

## Appendix A

It is worth mentioning that the lifetime distribution at (0,τ) is just determined by the first phase model. In this way, we can easily obtain the expression of lifetime at (0,τ) directly as shown in (9) based on the property of the Wiener process.

As discussed before, what should be noticed is that lifetime distribution at the change point is not continuous, it can also be defined as the failure probability i.e., $\mathrm{Pr}\left\{x_{\tau^{-}}+\gamma \geq \xi \right\}$. If the jump is negative, the degradation process will not reach the threshold, i.e., failure probability at the change point is 0. In this way, we mainly focus on the positive case. On contrary, if the jump is not negative, we can have,

(A1) $$\begin{array}{cc} & \mathrm{Pr}\left(T=\tau \right)=\mathrm{Pr}\left(x_{\tau }=x_{\tau^{-}}+\gamma \geq \xi \right)\\ = & \mathrm{Pr}(x_{\tau^{-}}\geq \xi -\gamma )\\ = & \int_{\xi -\gamma }^{+\infty }g_{\tau^{-}}\left(x_{\tau^{-}}\right)dx_{\tau^{-}}\\ = & \int_{\xi -\gamma }^{\xi }g_{\tau^{-}}\left(x_{\tau^{-}}\right)dx_{\tau^{-}}\\ = & \int_{\xi -\gamma }^{\xi }\frac{1}{\sqrt{2\pi \tau^{-}\sigma_{1}^{2}}}\left\{\exp\left[-\frac{{\left(x_{\tau^{-}}-\mu_{1}\tau^{-}\right)}^{2}}{2\sigma_{1}^{2}\tau^{-}}\right]\right.\left.-\exp\left(\frac{2\mu_{1}\xi }{\sigma_{1}^{2}}\right)\exp\left[-\frac{{\left(x_{\tau^{-}}-2\xi -\mu_{1}\tau^{-}\right)}^{2}}{2\sigma_{1}^{2}\tau^{-}}\right]\right\}dx_{\tau^{-}} \end{array}$$

Since $x_{\tau^{-}}$ belongs to (−∞,ξ), $\int_{\xi -\gamma }^{+\infty }g_{\tau^{-}}\left(x_{\tau^{-}}\right)dx_{\tau^{-}}=\int_{\xi -\gamma }^{\xi }g_{\tau^{-}}\left(x_{\tau^{-}}\right)dx_{\tau^{-}}$. Then, according to property of truncated Gaussian distribution, we can solve the above integral with two parts,

(A2) $$\begin{array}{ccc} \mathrm{Pr}(T=\tau ) & = & \mathrm{Pr}(x_{\tau }=x_{\tau^{-}}+\gamma \geq \xi )\\ & = & \left[\Phi \left(\frac{\mu_{1}t-\xi }{\sigma \sqrt{t}}\right)-\exp\left(\frac{2\xi \mu_{1}}{\sigma }\right)\Phi \left(\frac{-\mu_{1}t-\xi }{\sigma \sqrt{t}}\right)\right]\\ & & +\left[-\Phi \left(\frac{\mu_{1}t-\xi +\gamma }{\sigma \sqrt{t}}\right)+\exp\left(\frac{2\left(\xi -\gamma \right)-\mu_{1}}{\sigma_{1}}\right)\Phi \left(\frac{-\mu_{1}t-\xi +\gamma }{\sigma_{1}\sqrt{t}}\right)\right] \end{array}$$

Next, we try to derive the lifetime distribution at (τ,+∞). The key to obtain the lifetime distribution at (τ,+∞) is the expression of $x_{\tau }$. Since $x_{\tau }=x_{\tau^{-}}+\gamma$, the PDF of $x_{\tau }$ can have $g_{\tau }\left(x_{\tau }\right)=g_{\tau^{-}}\left(x_{\tau }-\gamma \right)$, where $x_{\tau }\in \left(-\infty ,\tilde{\xi }\right)$ and $\tilde{\xi }=\xi -I\left(\gamma \leq 0\right)\cdot \gamma$. Then according to the law of total probability, the lifetime distribution at (τ,+∞) can be derived as,

(A3) $$\begin{array}{c} f_{T}\left(t\right)=\int_{-\infty }^{\tilde{\xi }}\frac{\xi -x_{\tau }}{\sqrt{2\pi \sigma_{2}^{2}{\left(t-\tau \right)}^{3}}}\exp\left[-\frac{{\left(\xi -x_{\tau }-\mu_{2}\left(t-\tau \right)\right)}^{2}}{2\sigma_{2}^{2}\left(t-\tau \right)}\right]g_{\tau }\left(x_{\tau }\right)dx_{\tau },\;\;t>\tau \end{array}$$

In this way, we have obtained the form of lifetime distribution at (τ,+∞) based on the property of Lemma 1 as shown (10), and thus the expression of the lifetime’s distribution for two-phase model with fixed parameters has been attained.

## Appendix B

To conduct the lifetime distribution with random drift coefficient, we can utilize the property of the law of total probability and linear Wiener process, and then its expression with single integral can be written as,

(A4) $$\begin{array}{c} f_{T}\left(t\right)=\int_{-\infty }^{+\infty }\frac{\xi -x_{0}}{\sqrt{2\pi \sigma_{1}^{2}t^{3}}}\exp\left[-\frac{{\left(\xi -x_{0}-\mu_{1}t\right)}^{2}}{2\sigma_{1}^{2}t}\right]\frac{1}{\sqrt{2\pi \sigma_{1p}^{2}}}\exp\left[-\frac{{\left(\mu_{1}-\mu_{1p}\right)}^{2}}{2\sigma_{1p}^{2}}\right]d\mu_{1},\;\;\;0<t\leq \tau \end{array}$$

It is noteworthy that the above integral form is similar to the expression in Lemma 2, then we can solve the above integral based on Lemma 2,

(A5) $$\begin{array}{c} f_{T}\left(t\right)=\frac{\xi -x_{0}}{\sqrt{2\pi t^{2}\left(t\sigma_{1}^{2}+t^{2}\sigma_{1p}^{2}\right)}}\exp\left[-\frac{{\left(\xi -x_{0}-\mu_{1p}t\right)}^{2}}{2(t\sigma_{1}^{2}+t^{2}\sigma_{1p}^{2})}\right],\;\;\;0<t\leq \tau \end{array}$$

In this way, the proof has been completed.

## Appendix C

Firstly, we should try to conduct the PDF of $x_{\tau^{-}}$ with random effect. Based on the law of total probability, the PDF of $x_{\tau^{-}}$ with random effect can be rewritten by PDF of $x_{\tau^{-}}$ without random effect as shown in following equation,

(A6) $$\begin{array}{c} g_{\tau^{-}}\left(x_{\tau^{-}}|\mu_{1p},\sigma_{1p}\right)=\int_{-\infty }^{+\infty }\frac{g_{\tau^{-}}\left(x_{\tau^{-}}|\mu_{1}\right)}{\sqrt{2\pi \sigma_{1p}^{2}}}\exp\left[-\frac{{\left(\mu_{1}-\mu_{1p}\right)}^{2}}{2\sigma_{1p}^{2}}\right]d\mu_{1}\\ \int_{\xi -\gamma }^{\xi }\frac{1}{\sqrt{2\pi \tau^{-}\sigma_{1}^{2}}}\left\{\exp\left[-\frac{{\left(x_{\tau^{-}}-\mu_{1}\tau^{-}\right)}^{2}}{2\sigma_{1}^{2}\tau^{-}}\right]\right.\left.-\exp\left(\frac{2\mu_{1}\xi }{\sigma_{1}^{2}}\right)\exp\left[-\frac{{\left(x_{\tau^{-}}-2\xi -\mu_{1}\tau^{-}\right)}^{2}}{2\sigma_{1}^{2}\tau^{-}}\right]\right\}dx_{\tau^{-}}\\ =\left[1-\exp\left(-\frac{4\xi^{2}-4x_{\tau^{-}}\xi }{2\sigma_{1}^{2}\tau }\right)\right]\frac{1}{\sqrt{2\pi (\tau \sigma_{1}^{2}+\tau^{2}\sigma_{1p}^{2})}}\exp\left[-\frac{{\left(x_{\tau^{-}}-\mu_{1p}\tau \right)}^{2}}{2(\tau \sigma_{1}^{2}+\tau^{2}\sigma_{1p}^{2})}\right]\\ =\frac{\exp\left[-\frac{{\left(x_{\tau^{-}}-\mu_{1p}\tau \right)}^{2}}{2(\tau \sigma_{1}^{2}+\tau^{2}\sigma_{1p}^{2})}\right]-\exp\left(\frac{2\mu_{1p}\xi }{\sigma_{1}^{2}}+\frac{2(\xi^{2}\sigma_{1p}^{4}\tau +\xi^{2}\sigma_{1p}^{2}\sigma_{1}^{2})}{\left(\sigma_{1}^{2}+\tau \sigma_{1p}^{2}\right)\sigma_{1}^{4}}\right)\exp\left[-\frac{{\left(x_{\tau^{-}}-2\xi -\mu_{1p}\tau -\frac{2\xi \sigma_{1p}^{2}\tau }{\sigma_{1}^{2}}\right)}^{2}}{2(\tau \sigma_{1}^{2}+\tau^{2}\sigma_{1p}^{2})}\right]}{\sqrt{2\pi (\tau \sigma_{1}^{2}+\tau^{2}\sigma_{1p}^{2})}} \end{array}$$

Then because of $x_{\tau }=x_{\tau^{-}1}+\gamma$ and $\gamma ∼N(\mu_{\gamma },\sigma_{\gamma }^{2})$, we can obtain,

(A7) $$\begin{array}{c} g_{\tau }\left(x_{\tau }\right)=\int g_{\tau^{-}}\left(x_{\tau -\gamma }|\mu_{1p},\sigma_{1p}\right)p\left(\gamma \right)d\gamma \end{array}$$

where

(A8) $$\begin{array}{c} p\left(\gamma \right)=\frac{1}{\sqrt{2\pi \sigma_{\gamma }^{2}}}\exp\left[\frac{{\left(\gamma -\mu_{\gamma }\right)}^{2}}{2\sigma_{\gamma }^{2}}\right] \end{array}$$

Then, according Lemma 1, the $g_{\tau }\left(x_{\tau }\right)$ can be obtained,

(A9) $$\begin{array}{cc} g_{\tau }\left(x_{\tau }\right)= & \sqrt{\frac{1}{2\pi \left(\sigma_{a}^{2}+\sigma_{b}^{2}\right)}}\exp\left(-\frac{{\left(\mu_{a}-\mu_{b}\right)}^{2}}{2\left(\sigma_{a}^{2}+\sigma_{b}^{2}\right)}\right)\left[1-\Phi \left(-\frac{\xi \left(\sigma_{a}^{2}+\sigma_{b}^{2}\right)-\mu_{b}\sigma_{a}^{2}-\mu_{a}\sigma_{b}^{2}}{\sqrt{\sigma_{a}^{2}\sigma_{b}^{2}\left(\sigma_{a}^{2}+\sigma_{b}^{2}\right)}}\right)\right]\\ & -\exp\left(\frac{2\mu_{1p}\xi }{\sigma_{1}^{2}}+\frac{2\xi^{2}\sigma_{1p}^{2}}{\sigma_{1}^{4}}\right)\sqrt{\frac{1}{2\pi \left(\sigma_{a}^{2}+\sigma_{c}^{2}\right)}}\exp\left(-\frac{{\left(\mu_{a}-\mu_{c}\right)}^{2}}{2\left(\sigma_{a}^{2}+\sigma_{c}^{2}\right)}\right)\\ & \times \left[1-\Phi \left(-\frac{\xi \left(\sigma_{a}^{2}+\sigma_{c}^{2}\right)-\mu_{c}\sigma_{a}^{2}-\mu_{a}\sigma_{c}^{2}}{\sqrt{\sigma_{a}^{2}\sigma_{c}^{2}\left(\sigma_{a}^{2}+\sigma_{c}^{2}\right)}}\right)\right] \end{array}$$

where

(A10) $$\begin{array}{c} \begin{array}{c} \mu_{a}=x_{\tau }-\mu_{\gamma },\;\mu_{b}=\mu_{1p}\tau_{1},\;\mu_{c}=\mu_{1p}\tau_{1}+2\xi +\frac{\sigma_{1p}^{2}\tau }{\sigma_{1}^{2}}\\ \sigma_{a}=\sigma_{\gamma },\;\sigma_{b}=\sigma_{c}=\sqrt{\tau_{1}\sigma_{1}^{2}+\tau_{1}^{2}\sigma_{1p}^{2}},\; \end{array} \end{array}$$

## Appendix D

For simplicity, we ignore the information of $x_{{\tilde{\tau }}_{i}+1}-x_{{\tilde{\tau }}_{i}}$ for parameters estimation based on the EM algorithm. Then, according to the EM algorithm, two steps should be included as follows,

E-step:

(A11) Q(Ξ|Ξ^(k))=EZ|X,Ξ^(k)lnp(X,Z|Ξ)=EZ|X,Ξ^(k)∑i=1nlnp(Zi|Ξ)p(Xi|Zi,Ξ)=∑i=1nEZi|Xi,Ξ^(k)lnp(Zi|Ξ)p(Xi|Zi,Ξ)=∑i=1nEZi|Xi,Ξ^(k)ln12πσ1p2exp−(μ1,i−μ1p)22σ1p2Πj=1τ˜i12πσ12Δtexp−(xi,j−xi,j−1−μ1,iΔt)22σ12Δt 12πσ2p2exp−(μ2,i−μ2p)22σ2p2Πj=τ˜i+2mi12πσ22Δtexp−(xi,j−xi,j−1−μ2,iΔt)22σ22Δt

where $Z_{i}=\left\{\mu_{1,i},\mu_{2,i}\right\}$. To conduct the above equation, we firstly the following conditional probability based on Bayesian rule.

(A12) $$\begin{array}{c} p\left(Z_{i}|X_{i},\Xi^{\left(k\right)}\right)=\frac{p\left(X_{i}|Z_{i},\Xi^{\left(k\right)}\right)p\left(Z_{i}|\Xi^{\left(k\right)}\right)}{\int p\left(X_{i}|Z_{i},\Xi^{\left(k\right)}\right)p\left(Z_{i}|\Xi^{\left(k\right)}\right)dZ_{i}} \end{array}$$

Thus, we can obtain the conditional probability $p(\mu_{1,i}|X_{i},\Xi^{\left(k\right)})$ and $p(\mu_{2,i}|X_{i},\Xi^{\left(k\right)})$ as follows,

(A13) $$\begin{array}{c} p\left(\mu_{1,i}|X_{i},\Xi^{\left(k\right)}\right)=\frac{1}{\sqrt{2\pi \frac{\sigma_{1p}^{2,(k)}\sigma_{1}^{2,(k)}\Delta t}{{\tilde{\tau }}_{i}\sigma_{1p}^{2,(k)}\Delta t^{2}+\sigma_{1}^{2,(k)}\Delta t}}}\exp\left[-\frac{{\left(\mu_{1,i}-\frac{\left(x_{i,{\tilde{\tau }}_{i}}-x_{i,0}\right)\Delta t\sigma_{1p}^{2,(k)}+\sigma_{1}^{2,(k)}\Delta t\mu_{1p}^{\left(k\right)}}{{\tilde{\tau }}_{i}\sigma_{1p}^{2,(k)}\Delta t^{2}+\sigma_{1}^{2,(k)}\Delta t}\right)}^{2}}{\frac{2\sigma_{1p}^{2,(k)}\sigma_{1}^{2,(k)}\Delta t}{{\tilde{\tau }}_{i}\sigma_{1p}^{2,(k)}\Delta t^{2}+\sigma_{1}^{2,(k)}\Delta t}}\right] \end{array}$$

(A14) $$\begin{array}{c} p\left(\mu_{2,i}|X_{i},\Xi^{\left(k\right)}\right)=\frac{1}{\sqrt{2\pi \frac{\sigma_{2p}^{2,(k)}\sigma_{2}^{2,(k)}\Delta t}{\left(m_{i}-{\tilde{\tau }}_{i}-1\right)\sigma_{2p}^{2,(k)}\Delta t^{2}+\sigma_{2}^{2,(k)}\Delta t}}}\exp\left[-\frac{{\left(\mu_{2,i}-\frac{\left(x_{i,m_{i}}-x_{i,{\tilde{\tau }}_{i}+1}\right)\Delta t\sigma_{2p}^{2,(k)}+\sigma_{2}^{2,(k)}\Delta t\mu_{2p}^{\left(k\right)}}{\left(m_{i}-{\tilde{\tau }}_{i}-1\right)\sigma_{2p}^{2,(k)}\Delta t^{2}+\sigma_{2}^{2,(k)}\Delta t}\right)}^{2}}{\frac{2\sigma_{2p}^{2,(k)}\sigma_{2}^{2,(k)}\Delta t}{\left(m_{i}-{\tilde{\tau }}_{i}-1\right)\sigma_{2p}^{2,(k)}\Delta t^{2}+\sigma_{2}^{2,(k)}\Delta t}}\right] \end{array}$$

Then it is noted that $p(\mu_{1,i}|X_{i},\Xi^{\left(k\right)})$ and $p(\mu_{2,i}|X_{i},\Xi^{\left(k\right)})$ are the PDFs of Gaussian distribution. Therefore, we can further attain the conditional expectation as show in (A11) based on the property of Gaussian distribution.

(A15) Q(Ξ|Ξ^(k))=∑i=1nEZi|Xi,Ξ^(k)lnp(Zi|Ξ)p(Xi|Zi,Ξ)=∑i=1nEZi|Xi,Ξ^(k)ln12πσ1p2exp−(μ1,i−μ1p)22σ1p2Πj=0τ˜i12πσ12Δtexp−(xi,j−xi,j−1−μ1,iΔt)22σ12Δt 12πσ2p2exp−(μ2,i−μ2p)22σ2p2Πj=τ˜i+2mi12πσ22Δtexp−(xi,j−xi,j−1−μ2,iΔt)22σ22Δt=nln12πσ1p2−∑i=1nE[μ1,i2|Xi,Ξ^(k)]−2μ1p∑i=1nE[μ1,i|Xi,Ξ^(k)]+nμ1p22σ2p2+∑i=1nτ˜iln12πσ12Δt −∑i=1n∑j=1τ˜i(xi,j−xi,j−1)2−2E[μ1,i|Xi,Ξ^(k)]∑j=1τ˜i(xi,j−xi,j−1)+τ˜iE[μ1,i2|Xi,Ξ^(k)]2σ12Δt nln12πσ2p2−∑i=1nE[μ2,i2|Xi,Ξ^(k)]−2μ2p∑i=1nE[μ2,i|Xi,Ξ^(k)]+nμ2p22σ2p2+∑i=1n(mi−τ˜i−1)ln12πσ22Δt −∑i=1n∑j=τ˜i+2mi(xi,j−xi,j−1)2−2E[μ2,i|Xi,Ξ^(k)]∑j=τ˜i+2mi(xi,j−xi,j−1)+(mi−τ˜i−1)E[μ2,i2|Xi,Ξ^(k)]2σ22Δt

where

(A16) $$\begin{array}{cc} E[\mu_{1,i}|X_{i},{\hat{\Xi }}^{\left(k\right)}]= & \frac{\left(x_{i,{\tilde{\tau }}_{i}}-x_{i,0}\right)\Delta t\sigma_{1p}^{2,(k)}+\sigma_{1}^{2,(k)}\Delta t\mu_{1p}^{\left(k\right)}}{{\tilde{\tau }}_{i}\sigma_{1p}^{2,(k)}\Delta t^{2}+\sigma_{1}^{2,(k)}\Delta t}\\ E[\mu_{1,i}^{2}|X_{i},{\hat{\Xi }}^{\left(k\right)}]= & \frac{\sigma_{1p}^{2,(k)}\sigma_{1}^{2,(k)}\Delta t}{{\tilde{\tau }}_{i}\sigma_{1p}^{2,(k)}\Delta t^{2}+\sigma_{1}^{2,(k)}\Delta t}+{\left[\frac{\left(x_{i,{\tilde{\tau }}_{i}}-x_{i,0}\right)\Delta t\sigma_{1p}^{2,(k)}+\sigma_{1}^{2,(k)}\Delta t\mu_{1p}^{\left(k\right)}}{{\tilde{\tau }}_{i}\sigma_{1p}^{2,(k)}\Delta t^{2}+\sigma_{1}^{2,(k)}\Delta t}\right]}^{2}\\ E[\mu_{2,i}|X_{i},{\hat{\Xi }}^{\left(k\right)}]= & \frac{\left(x_{i,m_{i}}-x_{i,{\tilde{\tau }}_{i}-1}\right)\Delta t\sigma_{2p}^{2,(k)}+\sigma_{2}^{2,(k)}\Delta t\mu_{2p}^{\left(k\right)}}{\left(m_{i}-{\tilde{\tau }}_{i}-1\right)\sigma_{2p}^{2,(k)}\Delta t^{2}+\sigma_{2}^{2,(k)}\Delta t}\\ E[\mu_{2,i}^{2}|X_{i},{\hat{\Xi }}^{\left(k\right)}]= & \frac{\sigma_{2p}^{2,(k)}\sigma_{2}^{2,(k)}\Delta t}{\left(m_{i}-{\tilde{\tau }}_{i}-1\right)\sigma_{2p}^{2,(k)}\Delta t^{2}+\sigma_{2}^{2,(k)}\Delta t}+{\left[\frac{\left(x_{i,m_{i}}-x_{i,{\tilde{\tau }}_{i}-1}\right)\Delta t\sigma_{2p}^{2,(k)}+\sigma_{2}^{2,(k)}\Delta t\mu_{2p}^{\left(k\right)}}{\left(m_{i}-{\tilde{\tau }}_{i}-1\right)\sigma_{2p}^{2,(k)}\Delta t^{2}+\sigma_{2}^{2,(k)}\Delta t}\right]}^{2} \end{array}$$

M-step: In order obtain the $\hat{\Xi }=\underset{\Xi }{argmax}Q\left(\Xi |{\hat{\Xi }}^{\left(k\right)}\right)$, we take the partial derivative of $\frac{\partial Q(\Xi |{\hat{\Xi }}^{\left(k\right)})}{\partial \Xi }$ and let $\frac{\partial Q(\Xi |{\hat{\Xi }}^{\left(k\right)})}{\partial \Xi }=0$. Then solve such the equation $\frac{\partial Q(\Xi |{\hat{\Xi }}^{\left(k\right)})}{\partial \Xi }=0$, the (k+1)-th step estimates of all parameters can be derived as follows,

(A17) $$\begin{array}{cc} {\hat{\mu }}_{1p}^{\left(k+1\right)}= & \frac{1}{n}\sum_{i=1}^{n}\frac{\left(x_{i,{\tilde{\tau }}_{i}}-x_{i,0}\right)\Delta t\sigma_{1p}^{2,(k)}+\sigma_{1,i}^{2,(k)}\Delta t\mu_{1p}^{\left(k\right)}}{{\tilde{\tau }}_{i}^{2}\sigma_{1p}^{2,(k)}\Delta t^{2}+\sigma_{1,i}^{2,(k)}\Delta t}\\ {\hat{\sigma }}_{1p}^{\left(k+1\right)}= & \sqrt{\frac{1}{n}\sum_{i=1}^{n}\left(E\left[\mu_{1,i}^{2}|X_{i},{\hat{\Xi }}^{\left(k\right)}\right]-E^{2}\left[\mu_{1,i}|X_{i},{\hat{\Xi }}^{\left(k\right)}\right]\right)}\\ {\hat{\mu }}_{2p}^{\left(k+1\right)}= & \frac{1}{n}\sum_{i=1}^{n}\frac{\left(x_{i,m_{i}}-x_{i,{\tilde{\tau }}_{i}-1}\right)\Delta t\sigma_{2p}^{2,(k)}+\sigma_{2,i}^{2,(k)}\Delta t\mu_{2p}^{\left(k\right)}}{{\left(m_{i}-{\tilde{\tau }}_{i}-1\right)}^{2}\sigma_{2p}^{2,(k)}\Delta t^{2}+\sigma_{2,i}^{2,(k)}\Delta t}\\ {\hat{\sigma }}_{2p}^{\left(k+1\right)}= & \sqrt{\frac{1}{n}\sum_{i=1}^{n}\left(E\left[\mu_{2,i}^{2}|X_{i},{\hat{\Xi }}^{\left(k\right)}\right]-E^{2}\left[\mu_{2,i}|X_{i},{\hat{\Xi }}^{\left(k\right)}\right]\right)}\\ {\hat{\sigma }}_{1}^{\left(k+1\right)}= & \sqrt{\frac{\sum_{i=1}^{n}\left\{\sum_{j=1}^{{\tilde{\tau }}_{i}}{\left(x_{i,j}-x_{i,j-1}\right)}^{2}-2E\left[\mu_{1,i}|X_{i},{\hat{\Xi }}^{\left(k\right)}\right]\sum_{j=1}^{{\tilde{\tau }}_{i}}\left(x_{i,j}-x_{i,j-1}\right)+{\tilde{\tau }}_{i}E\left[\mu_{1,i}^{2}|X_{i},{\hat{\Xi }}^{\left(k\right)}\right]\right\}}{\sum_{i=1}^{n}{\tilde{\tau }}_{i}\Delta t}}\\ {\hat{\sigma }}_{2}^{\left(k+1\right)}= & \sqrt{\frac{\sum_{i=1}^{n}\left\{\sum_{j={\tilde{\tau }}_{i}+2}^{m_{i}}{\left(x_{i,j}-x_{i,j-1}\right)}^{2}-2E\left[\mu_{2,i}|X_{i},{\hat{\Xi }}^{\left(k\right)}\right]\sum_{j={\tilde{\tau }}_{i}+2}^{m_{i}}\left(x_{i,j}-x_{i,j-1}\right)+\left(m_{i}-{\tilde{\tau }}_{i}-1\right)E\left[\mu_{2,i}^{2}|X_{i},{\hat{\Xi }}^{\left(k\right)}\right]\right\}}{\sum_{i=1}^{n}\left(m_{i}-{\tilde{\tau }}_{i}-1\right)\Delta t}} \end{array}$$

Thus, the derivation process has been completed.
